# Predicting worsening visual field via masked dual stage attention recurrent neural network

**DOI:** 10.1038/s41598-026-51152-3

**Published:** 2026-04-30

**Authors:** Keunheung Park, Hwayeong Kim, Sangwoo Moon, Junglim Kim, Sangwook Jin, Seunguk Lee, EunAh Kim, Jiwoong Lee

**Affiliations:** 1 Department of Ophthalmology, Busan Medical Center, Busan, Korea; 2 Department of Ophthalmology, Pusan National University College of Medicine, Busan, Korea; 3 Department of Ophthalmology, Yangsan Pusan National University College of Medicine, Yangsan, Korea; 4 Department of Ophthalmology, Busan Paik Hospital, Inje University College of Medicine, Busan, Korea; 5 Department of Ophthalmology, Dong-A University College of Medicine, Busan, Korea; 6 Department of Ophthalmology, Kosin University College of Medicine, Busan, Korea; 7 Department of Ophthalmology, Samsung Changwon Hospital, Sungkyunkwan University School of Medicine, Changwon, Korea; 8 Biomedical Research Institute, Pusan National University Hospital, Busan, Korea

**Keywords:** Visual field, Deep learning, Recurrent neural network, Attention, Eye diseases, Optic nerve diseases, Vision disorders

## Abstract

Based on dual-stage attention recurrent neural network (DA-RNN), a masked DA-RNN (MDA-RNN) model was developed to predict future visual fields (VF). The training dataset comprised 154,105 VF examinations of 22,404 eyes. Performance was measured as the mean absolute error (MAE) between the predicted and actual values of mean deviation (MD), pattern standard deviation (PSD), Visual Field Index (VFI), 54 values of total deviation values (TDV) according to glaucoma severity and the prediction interval. Attention weights were analyzed to determine the characteristics of the MDA-RNN. Prediction errors of MDA-RNN for MD, PSD, VFI, TDV were 1.44 ± 2.05 dB, 1.03 ± 1.09 dB, 4.19 ± 7.07%, and 2.45 ± 1.97 dB respectively. Except for the VFI error, MDA-RNN exhibited a significantly lower prediction error than the masked bidirectional gated recurrent unit (BiGRU) model. The MDA-RNN assigned large weights to the TDV, PDV, MD, and false negative ratio (FN) in the encoder attention. In decoder attention, high scores were assigned to VF with lower FN and higher MD. MDA-RNN focused more on important features and could select more reliable VFs over all time steps, reducing noise in the VF input. The MDA-RNN was robust in predicting severe glaucoma, largely worsening glaucoma, and offered long-term prediction intervals.

**Supplementary Information:**

The online version contains supplementary material available at 10.1038/s41598-026-51152-3.

## Introduction

Glaucoma, diabetic retinopathy, and age-related macular degeneration are among the three major causes of blindness^1^. Glaucoma is characterized by an irreversible and progressive loss of retinal ganglion cells and their axons. The patient’s visual field (VF) gradually narrows before losing total vision^2^. Detecting and monitoring glaucoma progression is important for preserving the patient’s vision. However, VF tests contain many errors and fluctuations, and it is difficult to determine whether VF is progressing^3^.

Extensive research has been conducted to predict future VF progression in glaucoma patients, evolving from traditional statistical models toward advanced time-series deep learning and generative frameworks. Traditional mathematical approaches primarily include Pointwise Linear Regression (PLR)^4^ and Variational Bayes Linear Regression (VBLR)^5^. More recently, time-series deep learning models, such as those utilizing Convolutional Long Short-Term Memory (ConvLSTM)^6^, have been developed. Furthermore, in the field of generative modeling, Wen et al.^7^ introduced the CascadeNet-5 model, which successfully achieved pointwise prediction of VF test results up to 5.5 years into the future. Moreover, recent machine learning frameworks have expanded broader glaucoma analysis, such as PyGlaucoMetrics, which unifies multiple visual field diagnostic criteria^8^. While these deep learning techniques outperformed conventional mathematical and other machine learning methods, they still did not account for the reliability of the input visual field tests.

The authors have also been dedicated to developing deep learning models for predicting future visual field progression. The first attempt was a simple long short term memory (LSTM) model to predict future VF using a fixed number of input VFs^9^. The second attempt employed a bidirectional gated recurrent unit (BiGRU) model, which achieved improved performance compared to the previous LSTM model^10^. However, the BiGRU model has limitations in that the number of input VF examinations was fixed. The authors developed a third model, BiGRU, using a masking technique (masked BiGRU) to overcome these shortcomings^11^. This masked BiGRU model accepts a variable number of VF inputs and showed better performance.

A new state-of-the-art architecture called transformer^12^ was developed in 2017. The transformer, initially designed for natural language processing (NLP), has a huge impact on the deep learning field and has begun to replace existing architectures, recurrent neural network (RNN), and convolutional neural networks (CNN). The main idea used in a transformer is the “attention mechanism” which allows the model to identify the most relevant parts of the input sequence and assign “attention weights” according to their importance.

The dual-stage attention recurrent neural network (DA-RNN)^13^ was introduced in 2017, a few months before the transformer architecture emerged. The DA-RNN was designed to make non-linear time-series predictions, such as the stock market. It is based on an RNN such as LSTM or a gated recurrent unit (GRU); however, the difference is that, similar to the transformer, it employs a two-stage attention mechanism in both the encoder and decoder. In the first stage, the attention mechanism extracts the relevant driving series of the input features. In the second stage, the attention mechanism determines the relevant encoder output across all time steps. These two attention mechanisms allowed the model to focus on more relevant features in each input vector and important sequences throughout all input vectors. The DA-RNN outperformed the numerical regression models and the existing RNN model, including RNN with one-stage attention architectures.

VF is nonlinear time-series data and contains many features in each examination. However, each VF examination can contain numerous errors, whereas a series of VF can contain numerous unreliable tests. To predict future VF more accurately, a deep-learning model that discriminates between more relevant features and reliable VF tests is required. We found that these characteristics of the VF examination were consistent with the concepts introduced in the DA-RNN. This led us to develop a Masked DA-RNN (MDA-RNN), which is a modified DA-RNN that employs a mask technique to accept a variable number of VF examinations per patient.

The primary objective of predicting future VF is to preemptively identify whether a patient’s condition will deteriorate. While many mathematical approaches currently exist to assess VF progression, they are often limited in their ability to account for the non-linear dynamics of glaucoma and the inherent subjectivity of VF testing^6^. By providing accurate and visualizable predictions of future VF outcomes, clinicians can gain a clear, intuitive understanding of how and where visual loss is occurring, which significantly facilitates the establishment of optimal treatment strategies. Moreover, integrating predicted results with actual VF data through mathematical analysis enables more precise verification of statistically significant changes.

This study aimed to develop a new deep learning architecture, the MDA-RNN to predict future VF and evaluated its performance in comparison with our previously masked BiGRU model. In addition, an in-depth analysis was conducted to determine the characteristics of attention values within the MDA-RNN and how they are associated with the VF test features.

## Results

The internal test dataset consisted of 16,642 VF examinations from 2166 eyes of 1161 participants. The demographic characteristics are shown in Table 1. The mean follow-up duration was 957 ± 878 days (2.61 ± 2.41 years) and the mean age was 52.6 ± 18.8 years old. The mean VF MD was − 6.32 ± 7.94 dB and mean prediction time interval was 224.2 ± 251.3 days (0.61 ± 0.69 years). The external test dataset consisted of 18,683 VF examinations from 2991 eyes of 1582 participants. Compared to the internal test set, the external test set was characterized by a higher mean age, longer average follow-up and prediction intervals, and a greater proportion of moderate-to-advanced glaucoma.

**Table 1:** Training and testing data demographic.

	Train set	Internal test set	External test set
No. of visual field exams	154,105	16,642	18,683
No. of eyes (patients)	22,404 (12,193)	2166 (1161)	2991 (1582)
Age (years)	57.1 ± 16.9	52.6 ± 18.8	61.0 ± 14.2
Average follow up period (days)	1766 ± 1,318	957 ± 878	2526 ± 1552
Visual field mean deviation (dB)	− 7.87 ± 8.75	− 6.32 ± 7.94	− 6.43 ± 5.87^a^
*No. of eyes according to glaucoma severity*			
Early glaucoma	13,275	1488	1919
Moderate glaucoma	3646	304	614
Advanced glaucoma	5483	374	458
*No. of eyes according to no. of input exams*			
3 ~ 5 exams	11,674	1006	2016
6 ~ 10 exams	6941	651	802
11 ~ 15 exams	2448	329	168
16 ~ 20 exams	922	117	5
21 ~ 30 exams	374	54	0
31 ~ exams	45	9	0
Mean prediction interval (days)	310.3 ± 334.6	224.2 ± 251.3	438.8 ± 285.1
*No. of eyes according to prediction interval*			
< 6 months	7446	953	276
6 ≤ ~ < 12 months	8765	881	648
12 ≤ ~ < 24 months	4533	235	1764
24 ≤ months	1660	97	303

^a^ Because the external dataset did not provide global index values, we used the mean total deviation—calculated as the arithmetic mean of the total deviation values (TDVs)—instead of Mean Deviation (MD), which is a standard global index.

The prediction performance on internal test set according to glaucoma severity is summarized in Table 2. Prediction errors of MDA-RNN for MD, PSD, VFI, TDV_P-MAE_, TDV_P-RMSE_ throughout all eyes were 1.44 ± 2.05 dB, 1.03 ± 1.09 dB, 4.19 ± 7.07%, 2.45 ± 1.97 dB, and 3.29 ± 2.40 dB, respectively. Except for VFI error (4.19% vs 4.17%), MDA-RNN showed significantly lower prediction error than masked BiGRU model. In early glaucoma, prediction errors of MDA-RNN are 1.04 ± 1.49 dB, 0.82 ± 0.86 dB, 2.39 ± 4.44%, 1.78 ± 1.33 dB, and 2.37 ± 1.58 dB (MD, PSD, VFI, TDV_P-MAE_, TDV_P-RMSE_, respectively) and all prediction errors are significantly lower than those of the masked BiGRU model. In moderate glaucoma, prediction errors of MDA-RNN are 2.01 ± 1.96 dB, 1.54 ± 1.39 dB, 6.33 ± 6.36%, 3.50 ± 1.55 dB, and 4.90 ± 2.02 dB (MD, PSD, VFI, TDV_P-MAE_, TDV_P-RMSE_, respectively) and prediction error between MDA-RNN and masked BiGRU is not significantly different. However, in advanced glaucoma, prediction errors of MDA-RNN are 2.60 ± 3.20 dB, 1.43 ± 1.35 dB, 9.59 ± 11.53%, 4.31 ± 2.72 dB, and 5.67 ± 2.98 dB (MD, PSD, VFI, TDV_P-MAE_, TDV_P-RMSE_, respectively) and PSD, TDV_P-MAE_, TDV_P-RMSE_ errors of MDA-RNN are significantly lower than those of masked BiGRU model.

**Table 2:** Prediction error on internal test set according to glaucoma severity.

Glaucoma severity	Model	Prediction errors				
		MD error (dB)	PSD error (dB)	VFI error (%)	TDV_P-MAE_ (dB)	TDV_P-RMSE_ (dB)
All eyes (n = 2166)	MDA-RNN	1.44 ± 2.05 (1.36, 1.53)	1.03 ± 1.09 (0.98, 1.07)	4.19 ± 7.07 (3.89, 4.49)	2.45 ± 1.97 (2.37, 2.54)	3.29 ± 2.40 (3.19, 3.39)
	BiGRU	1.48 ± 2.04 (1.39, 1.56)	1.21 ± 1.08 (1.16, 1.25)	4.17 ± 6.87 (3.89, 4.46)	2.56 ± 1.96 (2.48, 2.64)	3.39 ± 2.38 (3.29, 3.49)
	*P* value	0.042	< 0.001	0.002	< 0.001	< 0.001
Early glaucoma (n = 1488)	MDA-RNN	1.04 ± 1.49 (0.96, 1.11)	0.82 ± 0.86 (0.77, 0.86)	2.39 ± 4.44 (2.17, 2.62)	1.78 ± 1.33 (1.71, 1.84)	2.37 ± 1.58 (2.29, 2.45)
	BiGRU	1.10 ± 1.51 (1.02, 1.18)	1.02 ± 0.83 (0.97, 1.06)	2.46 ± 3.97 (2.26, 2.67)	1.89 ± 1.37 (1.82, 1.96)	2.48 ± 1.61 (2.39, 2.56)
	*P* value	< 0.001	< 0.001	< 0.001	< 0.001	< 0.001
Moderate glaucoma (n = 304)	MDA-RNN	2.01 ± 1.96 (1.79, 2.24)	1.54 ± 1.39 (1.38, 1.70)	6.33 ± 6.36 (5.61, 7.05)	3.50 ± 1.55 (3.32, 3.67)	4.90 ± 2.02 (4.67, 5.12)
	BiGRU	1.98 ± 1.81 (1.78, 2.19)	1.62 ± 1.36 (1.46, 1.77)	5.88 ± 5.60 (5.25, 6.51)	3.53 ± 1.50 (3.37, 3.70)	4.92 ± 1.98 (4.69, 5.14)
	*P* value	0.420	0.603	0.120	0.636	0.978
Advanced glaucoma (n = 374)	MDA-RNN	2.60 ± 3.20 (2.27, 2.92)	1.43 ± 1.35 (1.30, 1.57)	9.59 ± 11.53 (8.42, 10.77)	4.31 ± 2.72 (4.03, 4.59)	5.67 ± 2.98 (5.37, 5.97)
	BiGRU	2.56 ± 3.21 (2.24, 2.89)	1.62 ± 1.41 (1.48, 1.77)	9.60 ± 11.91 (8.39, 10.81)	4.43 ± 2.63 (4.17, 4.70)	5.78 ± 2.88 (5.48, 6.07)
	*P* value	0.947	0.011	0.947	< 0.001	0.003

Values are presented as mean ± standard deviation (95% C.I.; Hodges–Lehmann estimate). *P* value: Wilcoxon’s signed rank test between two AI models (Benjamini–Hochberg procedure applied to control the false discovery rate). MDA-RNN: (new model) Masked Dual stage Attention recurrent neural network. BiGRU: (previous model) Masked Bidirectional Gated Recurrent Unit. MD: Visual field mean deviation. PSD: Visual field pattern standard deviation. VFI: Visual Feld Index. TDV_P-MAE_: pointwise mean absolute error (MAE) of visual field total deviation values (TDV). TDV_P-RMSE_: pointwise root mean square error (RMSE) of visual field total deviation values (TDV).

The prediction error on internal test set according to the prediction interval (the time interval from the last VF examination to the future prediction) is also summarized in Table 3. For a prediction period of less than 6 months, except for VFI, all the prediction errors of the MDA-RNN are significantly lower than those of the masked BiGRU. For 6–12 months prediction period, MDA-RNN predicted PSD, TDV_P-MAE_, and TDV_P-RMSE_ more accurately than the masked BiGRU; however, MD and VFI errors do not significantly differ between the two models. For 12–24 months prediction period, the prediction errors of MDA-RNN are significantly lower than those of the masked BiGRU model, except for the MD error, with no significant difference between the two models. For predictions above 24 months, the MDA-RNN also predicted more accurately than the masked BiGRU model, except for MD, which did not significantly differ.

**Table 3:** Prediction error on internal test set according to prediction interval.

Prediction interval (months)	Model	Prediction errors				
		MD error (dB)	PSD error (dB)	VFI error (%)	TDV_P-MAE_ (dB)	TDV_P-RMSE_ (dB)
< 6 (n = 953)	MDA-RNN	1.58 ± 2.35 (1.43, 1.73)	1.13 ± 1.23 (1.05, 1.21)	4.75 ± 8.09 (4.24, 5.27)	2.61 ± 2.24 (2.47, 2.76)	3.51 ± 2.67 (3.34, 3.68)
	BiGRU	1.66 ± 2.23 (1.52, 1.80)	1.31 ± 1.20 (1.24, 1.39)	4.73 ± 7.53 (4.25, 5.21)	2.74 ± 2.13 (2.60, 2.87)	3.62 ± 2.56 (3.45, 3.78)
	*P* value	0.001	< 0.001	0.051	< 0.001	< 0.001
6 ≤ ~ < 12 (n = 881)	MDA-RNN	1.22 ± 1.37 (1.13, 1.31)	0.90 ± 0.88 (0.85, 0.96)	3.46 ± 4.59 (3.16, 3.77)	2.25 ± 1.41 (2.16, 2.34)	3.05 ± 1.88 (2.92, 3.17)
	BiGRU	1.22 ± 1.35 (1.13, 1.31)	1.05 ± 0.86 (0.99, 1.10)	3.36 ± 4.46 (3.07, 3.66)	2.33 ± 1.43 (2.23, 2.42)	3.11 ± 1.87 (2.99, 3.24)
	*P* value	0.967	< 0.001	0.384	< 0.001	< 0.001
12 ≤ ~ < 24 (n = 235)	MDA-RNN	1.78 ± 2.87 (1.41, 2.15)	1.04 ± 1.19 (0.89, 1.19)	4.80 ± 10.12 (3.50, 6.10)	2.65 ± 2.60 (2.32, 2.99)	3.43 ± 2.95 (3.05, 3.80)
	BiGRU	1.76 ± 3.09 (1.36, 2.16)	1.23 ± 1.07 (1.09, 1.37)	5.05 ± 10.81 (3.66, 6.43)	2.76 ± 2.81 (2.40, 3.12)	3.53 ± 3.11 (3.13, 3.93)
	*P* value	0.642	< 0.001	0.013	0.012	0.012
24 ≤ (n = 97)	MDA-RNN	1.32 ± 1.51 (1.02, 1.63)	1.08 ± 1.17 (0.84, 1.31)	3.76 ± 5.10 (2.74, 4.79)	2.26 ± 1.54 (1.95, 2.57)	3.09 ± 2.09 (2.67, 3.51)
	BiGRU	1.30 ± 1.70 (0.96, 1.65)	1.53 ± 1.33 (1.27, 1.80)	4.03 ± 4.80 (3.07, 5.00)	2.42 ± 1.73 (2.08, 2.77)	3.29 ± 2.30 (2.83, 3.76)
	*P* value	0.380	< 0.001	0.050	0.005	0.005

Values are presented as mean ± standard deviation (95% C.I.; Hodges–Lehmann estimate). *P* value: Wilcoxon’s signed rank test between two AI models (Benjamini–Hochberg procedure applied to control the false discovery rate). MDA-RNN: (new model) Masked Dual stage Attention recurrent neural network. BiGRU: (previous model) Masked Bidirectional Gated Recurrent Unit. MD: Visual field mean deviation. PSD: Visual field pattern standard deviation. VFI: Visual Feld Index. TDV_P-MAE_: pointwise mean absolute error (MAE) of visual field total deviation values (TDV). TDV_P-RMSE_: pointwise root mean square error (RMSE) of visual field total deviation values (TDV).

The prediction errors evaluated on the external test set are summarized in Tables 4 and 5. Unlike internal test set, the prediction errors for MD, PSD, and VFI were not presented because the actual values were not provided in the external dataset. For the MDA-RNN, the overall prediction errors for TDV_P-MAE_, and TDV_P-RMSE_ were 2.90 dB, and 3.79 dB, respectively. These values were slightly higher than those observed in the internal test set. The overall prediction errors of the masked BiGRU model were 3.26 dB (TDV_P-MAE_), and 4.13 dB (TDV_P-RMSE_) which were larger than those of MDA-RNN.

**Table 4:** Prediction error on external test set according to glaucoma severity.

Glaucoma severity	Model	Prediction errors	
		TDV_P-MAE_ (dB)	TDV_P-RMSE_ (dB)
All eyes (n = 2991	MDA-RNN	2.90 ± 2.02 (95% CI 2.82, 2.97)	3.79 ± 2.43 (95% CI 3.70, 3.87)
	BiGRU	3.26 ± 2.13 (95% CI 3.19, 3.34)	4.13 ± 2.46 (95% CI 4.05, 4.22)
	*P* value	< 0.001	< 0.001
Early glaucoma (n = 1919)	MDA-RNN	2.23 ± 1.63 (95% CI 2.15, 2.30)	2.89 ± 1.91 (95% CI 2.80, 2.97)
	BiGRU	2.70 ± 1.87 (95% CI 2.61, 2.78)	3.34 ± 2.05 (95% CI 3.25, 3.44)
	*P* value	< 0.001	< 0.001
Moderate glaucoma (n = 614)	MDA-RNN	3.73 ± 1.91 (95% CI 3.58, 3.88)	4.93 ± 2.24 (95% CI 4.75, 5.11)
	BiGRU	3.89 ± 2.15 (95% CI 3.72, 4.06)	5.06 ± 2.43 (95% CI 4.87, 5.25)
	*P* value	< 0.001	0.022
Advanced glaucoma (n = 458)	MDA-RNN	4.59 ± 2.21 (95% CI 4.39, 4.79)	6.01 ± 2.52 (95% CI 5.78, 6.24)
	BiGRU	4.78 ± 2.17 (95% CI 4.58, 4.98)	6.21 ± 2.45 (95% CI 5.98, 6.43)
	*P* value	< 0.001	< 0.001

Glaucoma severity group were defined by the patient’s last visual field mean deviation (MD) value. Early glaucoma is MD >  − 6 dB, Moderate glaucoma is − 6 ≥ MD  >  −12 dB, Advanced glaucoma is MD ≤  − 12 dB. Values are presented as mean ± standard deviation (95% C.I.; Hodges–Lehmann estimate). *P* value: Wilcoxon’s signed rank test between two AI models (Benjamini–Hochberg procedure applied to control the false discovery rate). MDA-RNN: (new model) Masked Dual stage Attention recurrent neural network. BiGRU: (previous model) Masked Bidirectional Gated Recurrent Unit. TDV_P-MAE_: pointwise mean absolute error (MAE) of visual field total deviation values (TDV). TDV_P-RMSE_: pointwise root mean square error (RMSE) of visual field total deviation values (TDV).

**Table 5:** Prediction error on external test set according to prediction interval.

Prediction interval	Model	Prediction errors	
		TDV_P-MAE_ (dB)	TDV_P-RMSE_ (dB)
< 6 (n = 276)	MDA-RNN	3.59 ± 2.21 (95% CI 3.32, 3.85)	4.69 ± 2.69 (95% CI 4.37, 5.01)
	BiGRU	3.81 ± 2.28 (95% CI 3.53, 4.08)	4.89 ± 2.68 (95% CI 4.57, 5.20)
	*P* value	< 0.001	< 0.001
6 ≤ ~ < 12 (n = 648)	MDA-RNN	3.06 ± 2.22 (95% CI 2.89, 3.23)	3.99 ± 2.62 (95% CI 3.79, 4.19)
	BiGRU	3.34 ± 2.32 (95% CI 3.17, 3.52)	4.27 ± 2.65 (95% CI 4.07, 4.47)
	*P* value	< 0.001	< 0.001
12 ≤ ~ < 24 (n = 1764)	MDA-RNN	2.77 ± 1.91 (95% CI 2.69, 2.86)	3.63 ± 2.28 (95% CI 3.52, 3.74)
	BiGRU	3.20 ± 2.05 (95% CI 3.10, 3.30)	4.04 ± 2.34 (95% CI 3.93, 4.15)
	*P* value	< 0.001	< 0.001
24 ≤ (n = 303)	MDA-RNN	2.64 ± 1.89 (95% CI 2.43, 2.85)	3.43 ± 2.33 (95% CI 3.17, 3.69)
	BiGRU	2.95 ± 1.97 (95% CI 2.73, 3.17)	3.72 ± 2.33 (95% CI 3.46, 3.99)
	*P* value	< 0.001	< 0.001

Values are presented as mean ± standard deviation (95% C.I.; Hodges–Lehmann estimate). *P* value: Wilcoxon’s signed rank test between two AI models (Benjamini–Hochberg procedure applied to control the false discovery rate). MDA-RNN: (new model) Masked Dual stage Attention recurrent neural network. BiGRU: (previous model) Masked Bidirectional Gated Recurrent Unit. TDV_P-MAE_: pointwise mean absolute error (MAE) of visual field total deviation values (TDV). TDV_P-RMSE_: pointwise root mean square error (RMSE) of visual field total deviation values (TDV).

Consistent with the findings from the internal test set, the prediction error according to glaucoma severity was smallest in early glaucoma and increased as the disease progressed to moderate and advanced stages. Regarding the prediction interval, the errors did not increase with longer time intervals; instead, they exhibited a tendency to decrease, which was also consistent with the results observed in the internal test set.

Furthermore, we evaluated the models’ performance on the internal test set by fixing the three reliability indices—false negative (FN) ratio, false positive (FP) ratio, and fixation loss (FL) ratio—at 15%, 15%, and 15%, respectively, to match the external test set. Under these conditions, the predictive performance of both models deteriorated; the prediction errors for MDA-RNN (not shown in table) increased to 1.52 dB (MD), 1.08 dB (PSD), 4.67% (VFI), 2.55 dB (TDV_P-MAE_), and 3.38 dB (TDV_P-RMSE_). Similarly, the masked BiGRU showed a decrease in performance, with errors rising to 1.70 dB (MD), 1.25 dB (PSD), 4.79% (VFI), 2.70 dB (TDV_P-MAE_), and 3.53 dB (TDV_P-RMSE_). These findings demonstrate that both the MDA-RNN and masked BiGRU models leverage reliability indices to enhance prediction accuracy. The absence or inaccuracy of these indices leads to a significant increase in prediction errors, highlighting their critical role in the models’ performance.

**Fig. 1:**
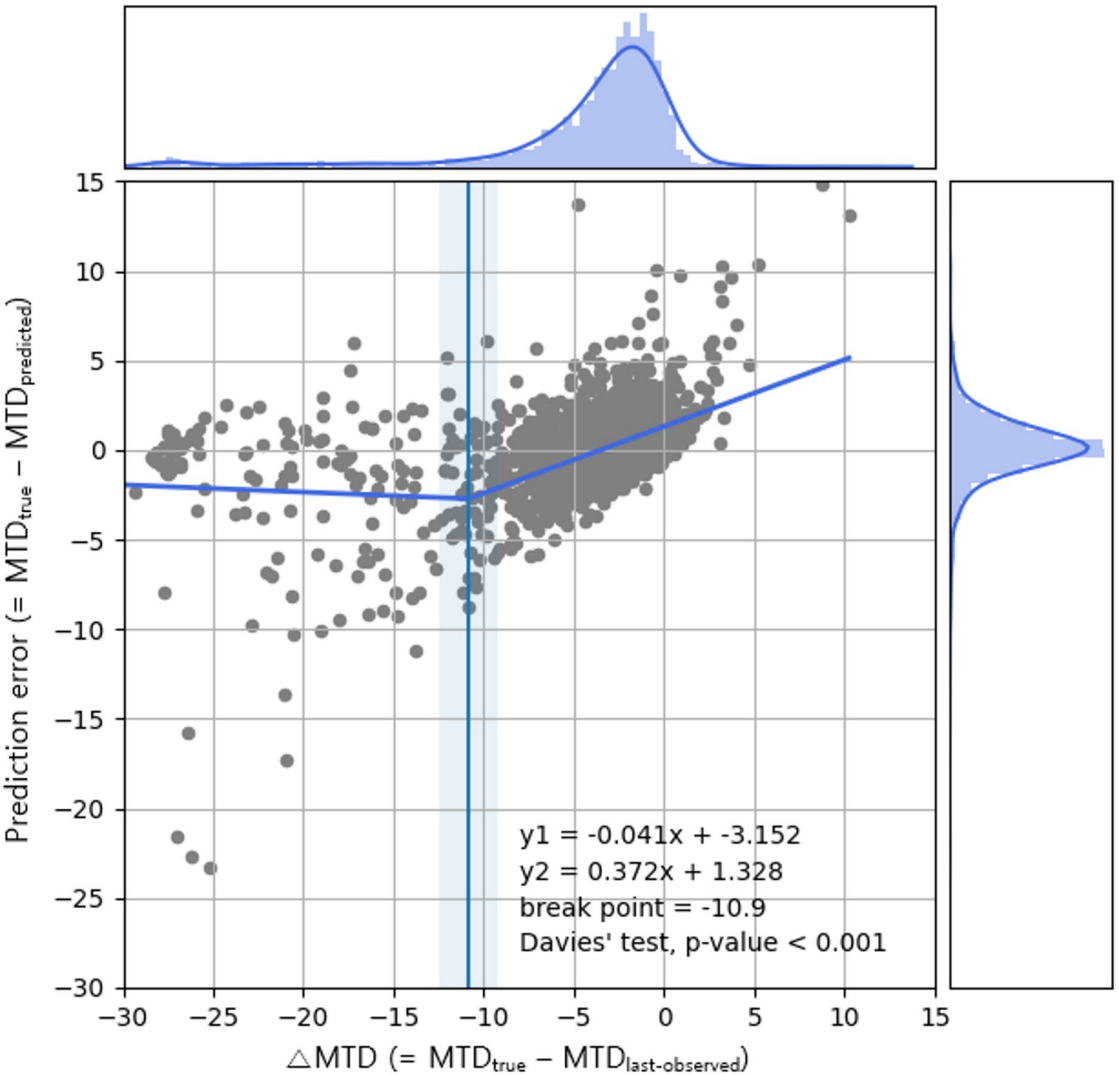
Scatterplot of visual field (VF) prediction error versus changes in mean total deviation value (MTD). MTD is arithmetic mean of the 54 total deviation values. Prediction error is MTD_true_–MTD_predicted_. △MTD represents changes in MTD between last observed and actual future VF. Initially, the prediction error negatively increases as the MTD becomes increasingly negative (slope = 0.372). However, after reaching a certain threshold value of MTD (breakpoint =  − 10.9 dB, Davies’ test *p* value < 0.001), the prediction error tends to decreases slightly (slope =  −  0.041).

MDA-RNN showed good performance, even in VF with substantial changes. Figure 1 shows the prediction error versus △MTD (mean total deviation). MTD is the arithmetic mean of the 54 total deviation values and △MTD (= MTD_true_ − MTD_last-observed_) is the difference between the last (most recent) observed MTD and the true MTD at the time of prediction. A higher absolute value of △MTD (x-axis) indicates a greater deviation in the VF; in particular, increasingly negative values signify more severe VF impairment. Initially, the prediction error negatively increases as the MTD becomes increasingly negative (slope = 0.372). However, after reaching a certain threshold value of MTD (breakpoint =  − 10.9 dB, Davies’ test *p* value < 0.001), the prediction error tends to decreases slightly (slope =  −  0.041).

**Fig. 2:**
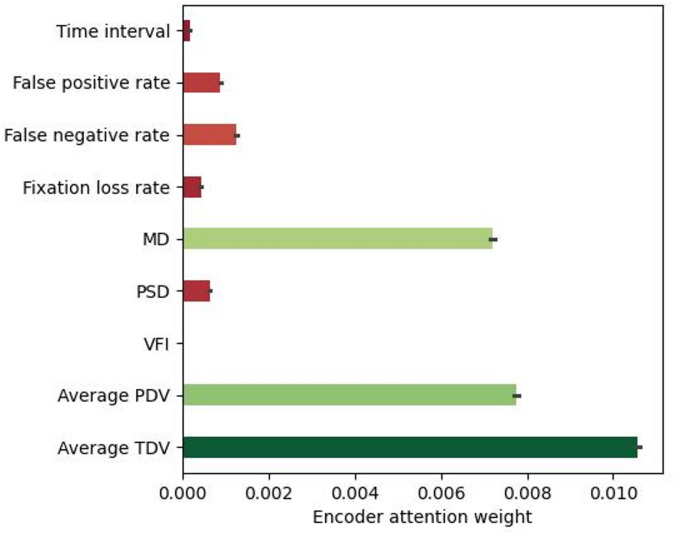
Barplot of encoder attention weights. MDA-RNN assigned large weights on TDV, PDV, and MD. Among reliability indices, MDA-RNN assigned the largest weight on false negative rate. *TDV* total deviation value, *PDV* pattern deviation value, *MD* visual field mean deviation, *PSD* pattern standard deviation, *VFI* visual field index. *MDA-RNN* masked dual-stage attention recurrent neural network.

Figure 2 shows the encoder attention weights for each input feature. The MDA-RNN encoder assigns large weights to TDV, PDV, and MD. Among the reliability indices, the false negative rate is assigned the greatest weight. Mean attention weight values ($\times {10}^{-3})$ are 10.56 ± 3.70, 7.76 ± 3.70, 7.19 ± 3.89 (TDV, PDV, MD, respectively) and 0.89 ± 1.17, 1.25 ± 1.91, 0.42 ± 0.97 (false positive rate (FP), false negative rate (FN), fixation loss rate (FL), respectively).

**Fig. 3:**
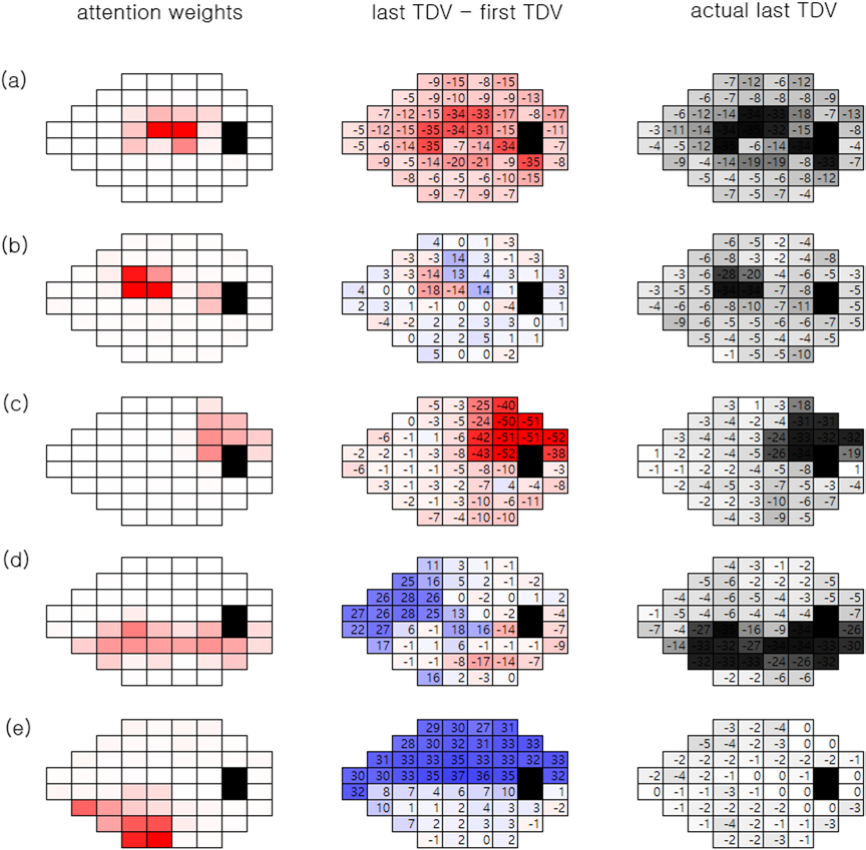
Pointwise analysis of encoder attention weights for total deviation value (TDV). Left column shows the strength of attention weights; darker red indicates higher weight. Middle column shows last (most recent) TDV—first visual field TDV. Red denotes negative change (worsened scotoma) and blue denotes positive change (improved scotoma). Right column shows actual last visual field exam. The darker region indicates more negative TDV (deeper scotoma). MDA-RNN assigned a larger attention weight to cases with a more negative difference (more worsened point in the middle column) and lower TDV point (more severe scotoma).

Figure 3 shows pointwise encoder attention weights and actual VF. The left column shows attention weights, where a more intense red means a higher weight. The middle column shows the pointwise TDV difference between the last and first input VF (most recent and initial exam, respectively), where red indicates a negative difference (worsened TDV), and blue indicates a positive difference (improved TDV). The right column shows pointwise actual TDV values of the last input VF; a darker shade indicates lower TDV. MDA-RNN assigns larger attention weight at points with a more negative difference (more worsened point in middle column) and lower TDV points (more severe scotoma). This is similar to the perspective of a human ophthalmologist who may focus on worsening points of TDV.

**Fig. 4:**
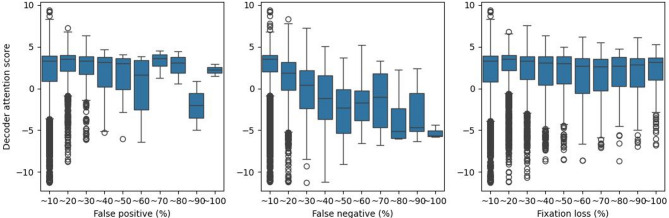
Boxplots of decoder attention score versus reliability indices. MDA-RNN assigned lower weights on visual field (VF) examination with a higher false negative rate, indicating that MDA-RNN concentrated on more reliable VFs. MDA-RNN did not respond to false positive or fixation loss rates.

Figure 4 shows the decoder attention score and reliability indices (false positive, false negative, and fixation loss rates). MDA-RNN assigns a low attention score as the false-negative rate increases. In other words, the MDA-RNN assigns less attention to low-reliability VF examinations. In contrast, MDA-RNN assigns similar attention weights regardless of false positive or fixation loss rate values.

**Fig. 5:**
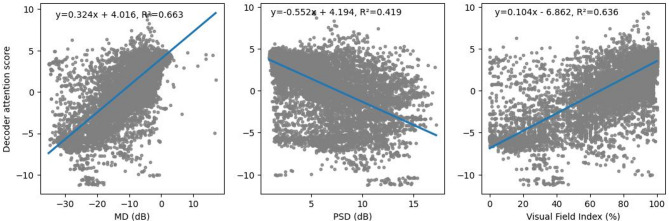
Scatterplots of decoder attention score versus global indices. Attention scores were strongly correlated with all global indices; MD and VFI were positively correlated, while PSD was negatively correlated. MDA-RNN tends to concentrate more on less severe visual field exams. *MD* visual field mean deviation, *PSD* pattern standard deviation, *VFI* visual field index. *MDA-RNN* masked dual-stage attention recurrent neural network.

The VF global indices (MD, PSD, and VFI) versus the decoder attention score are shown in Fig. 5. Decoder attention scores were strongly linearly correlated with all three global indices with R^2^ values of 0.663, 0.419, and 0.636 for MD, PSD, and VFI, respectively. The decoder attention score was positively correlated with MD and VFI (slope = 0.324 and 0.104, respectively) and negatively correlated with PSD (slope =  − 0.552), indicating that the MDA-RNN assigned lower scores to worse VF. The strength of the linear correlation was similar between MD and VFI, but that of PSD was relatively weaker.

## Discussion

MDA-RNN showed a significantly better performance than our previously masked BiGRU model. The overall prediction errors for MD, PSD, VFI, TDV_P-MAE_, TDV_P-RMSE_ were 1.44 ± 2.05 dB, 1.03 ± 1.09 dB, 4.19 ± 7.07%, 2.45 ± 1.97 dB, and 3.29 ± 2.40 dB, respectively. Even as the severity of glaucoma increased, MDA-RNN maintained a superior performance compared with that of the masked BiGRU model. Moreover, the performance gap between the two models widened further as the prediction interval increased. Even if the future VF was substantially changed compared with the input, the prediction error did not increase beyond a certain limit.

To ensure a more objective performance evaluation, this study utilized a large-scale external test dataset. The MDA-RNN achieved predictive performance of 2.90 dB (TDV_P-MAE_), and 3.79 dB (TDV_P-RMSE_), which was slightly lower than its performance on the internal test set. We attribute this slight decline to the absence of reliability index information in the external dataset, which necessitated fixing the false negative (FN), false positive (FP), and fixation loss (FL) values at 15%, 15%, and 15%, respectively. This constraint likely hindered the AI’s inherent mechanism for filtering out poor-quality visual field data and focusing on more reliable data points. This hypothesis is supported by our experiments on the internal test set, where fixing the reliability indices at 15/15/15 led to a deterioration in predictive performance for both the MDA-RNN and masked BiGRU. Unlike the internal test set, which excluded exams with reliability indices exceeding 33%, the external dataset had no such constraints. This potentially resulted in lower overall data quality; moreover, even the ground truth for the model’s output might have suffered from lower reliability, contributing to the larger observed errors. Additionally, the external test set comprised older patients and a higher proportion of moderate to advanced glaucoma cases. Despite these unfavorable conditions that inherently bias toward poorer outcomes, the MDA-RNN demonstrated robust results with only a marginal decrease in predictive accuracy.

Previous studies have consistently demonstrated that long-term test–retest variability of standard automated perimetry is substantial, particularly in glaucomatous eyes. Classic studies by Heijl et al.^14^ reported that pointwise threshold variability commonly ranges between 2 and 4 dB, increasing with defect depth and eccentricity. Similarly, Hutchings et al.^15^ showed that the 90–95% limits of agreement for between-visit fluctuation in stable glaucoma are approximately ± 3 dB, even in the absence of true progression. Comparable magnitudes of long-term variability were also reported by Boeglin et al.^16^ and Blumenthal et al.^17^, with global and pointwise fluctuations frequently exceeding 2 dB.

In this context, the MAE of our model for mean deviation (MD) prediction (1.44 dB) and total deviation values (TDV) (2.45 dB) falls within or below the expected range of physiological test–retest variability, indicating that the prediction error is comparable to the inherent noise of standard perimetric testing rather than representing clinically meaningful disagreement. Taken together, these comparisons indicate that the prediction errors of the proposed model are within the limits of normal perimetric variability and below established progression thresholds. Therefore, the model’s performance is unlikely to be confounded by measurement noise alone and may provide clinically useful information to assist in identifying patients who warrant closer surveillance or earlier therapeutic intervention.

In the internal test sets, the prediction errors for VFI were notably higher than those for MD and PSD. Because MD, PSD, and VFI are expressed on different scales (dB vs. percentage), direct comparison of absolute MAE values across metrics should be interpreted with caution. Furthermore, VFI incorporates a center-weighted non-linear calculation, making simple linear cross-metric comparisons inappropriate. Therefore, the prediction errors reported for each parameter should be interpreted independently within their respective clinical scales, rather than being compared directly against one another. When accounting for established equivalence between MD and VFI change rates^18^, the observed VFI MAE is consistent with clinically meaningful prediction accuracy.

The encoder attention in the MDA-RNN aimed to find more important features in each input vector. MDA-RNN assigned large weights to TDV, PDV, and MD because it was designed to predict 54 TDV values of VF. Moreover, a previous study reported that PDV tended to underestimate VF progression, and fewer progression were detected with PDV than that with TDV. Therefore, clinicians should monitor TDV and PDV to determine VF progression^19^. Encoder attention was thought to be well-trained, which is consistent with a previous study. It assigned a large weight to TDV and PDV but focused more on TDV. Among the reliability indices (FP, FN, and FL), MDA-RNN assigned a relatively large weight to FN, followed by FP and FL. This finding is consistent with those of previous studies^20–22^. Jithin et al.^20^ showed fixation loss had negligible impact on reliability in patients with established glaucoma. Harsha et al.^21^ emphasized the significant impact of FN in diagnosing glaucoma, even when the value is low, compared to that of FP and FL. Another study found that FN had the largest effect on MD, followed by FP, and FL appeared to have no association with MD^22^. MDA-RNN assigned weights in the same order as in the existing theory, and learned which VF examination is more reliable and needs to be focused.

In the pointwise analysis of encoder attention weights, MDA-RNN assigned relatively larger weights to the more negative TDV point and more negatively changed (worsened) TDV point and smaller weights to the positively changed (improved) TDV point. In other words, MDA-RNN focuses on the existing damaged and progressing points of the VF. This finding is consistent with the results of a previous study. Progressive VF locations do not occur randomly, but are most likely adjacent to the VF defects present in the initial tests^23^. Other studies have reported deepening of the existing scotoma as the most common pattern of VF progression (79%), followed by expansion (52%) and new scotomas (14%)^24,25^ Thus, focusing on existing scotomas may improve follow-up tests^24^.

Decoder attention in MDA-RNN is aimed selecting the relevant encoder output across all time steps. Humphrey’s VF examination is a subjective test that is largely influenced by the patient’s condition, but the results are highly variable. According to a previous study, VF performed in more than one-third of patients in a tertiary hospital was unreliable^26^. Another study recommended that patient reliability be considered when interpreting changes between VF examinations because VF results can vary depending on reliability^27^. Therefore, a well-designed decoder must be available to select a more reliable VF test result. In this study, the decoder attention of the MDA-RNN assigned a lower score to the VF with a higher FN, suggesting that the MDA-RNN focused more on reliable VF examinations. In addition, the decoder attention scores strongly correlated with all global indices. MD and VFI were positively correlated, and PSD was negatively correlated with the attention score. In a previous study, a worse baseline MD and higher FP and FN rates were associated with VF fluctuations^28^. Accordingly, MDA-RNN assigned lower attention scores to VFs with worse MD and higher FN. We concluded that the MDA-RNN learned from training to discriminate input VFs that are not reliable. Interestingly, among the global indices, PSD (R^2^ = 0.419) was less correlated with decoder attention weights than MD and VFI (R^2^ = 0.663, 0,636, respectively). We presume that this is because, unlike MD or VFI, which consistently decrease or increase as glaucoma progresses, PSD increases at first and then decreases^29^.

MDA-RNN performed well in predicting worsening VFs in patients with progressive glaucoma. Mohammad et al. evaluated two deep learning models based on convolutional neural networks (CNN) and RNN for potential biases in overestimating or underestimating VF changes over time and concluded that both models severely underpredict worsening of VF^30^. However, as shown in Fig. 1, the prediction error for MDA-RNN does not increase with worsening glaucoma. Even though the vast majority of the treated glaucoma patients remain stable over long time and approximately 3 ~ 17% of them are actually worsened,^31^ MDA-RNNs are well-trained to overcome this class imbalance problem.

The segmented regression observed in Fig. 1 suggests that prediction error increases with VF damage up to a certain point, consistent with known increases in test–retest variability. Interestingly, prediction error decreased beyond a △MTD of − 10.9. This may be partly explained by a floor effect in automated perimetry, which compresses the dynamic range of severely damaged test locations. Additionally, advanced glaucoma is characterized by spatially stable deepening of existing scotomas rather than random emergence of new defects^24,25^, potentially facilitating prediction. The attention mechanism of the MDA-RNN may further reduce error by focusing on consistently damaged and reliable VF locations. These factors together may account for the observed nonlinear relationship.

This study employed somewhat permissive reliability criteria for visual field tests, setting the thresholds for FP, FN, and FL at less than 33%. Although stricter reliability criteria are often recommended in clinical practice, applying such thresholds during model training may reduce data diversity and limit generalizability. In our approach, reliability indices were incorporated as model inputs, allowing the attention mechanism to selectively down-weight unreliable VF examinations rather than excluding them a priori.

Regarding the model inputs, age was not included as a separate variable because the MD, PSD, VFI, TDV, and PDV are inherently age-corrected. We aimed to focus the model’s learning capacity on the spatio-temporal patterns of vision loss itself, avoiding potential overfitting to chronological age. However, given that age is a critical risk factor for glaucoma progression, its inclusion in future iterations of the model could potentially provide further predictive insights.

It is important to note that the external test set differed from the training cohort in several aspects, including age, follow-up duration, and the distribution of glaucoma severity. Rather than performing matched-cohort adjustments, we intentionally evaluated our model on the unadulterated external dataset. Artificially matching cohorts could distort the natural distribution of the external clinical population and introduce selection bias. By demonstrating robust predictive performance on this distinct, unmodified dataset, we provide a fairer and more realistic representation of the model’s true external validity and its potential to generalize across heterogeneous real-world clinical environments.

A limitation of our study is the absence of glaucoma subtype stratification (e.g. primary open angle glaucoma, angle closure glaucoma, normal tension glaucoma). Because different glaucoma subtypes exhibit distinct patterns of visual field progression and rates of deterioration, disease heterogeneity could influence model training and predictive performance. Additionally, there are limitations regarding the handling of data imbalance, particularly for advanced glaucoma. Although we utilized external validation to help mitigate this issue, we did not perform advanced data balancing techniques such as reweighting, resampling, or calibration analysis. Future research should prioritize subtype-specific validation to ensure generalizability and incorporate comprehensive robustness analyses and calibration techniques to further optimize and validate model performance for severe disease stages.

MDA-RNN model offers substantial utility in clinical practice. By applying the model recursively, future changes can be presented period-by-period on a screen, integrated with existing VF test results for easy comparison. Furthermore, by utilizing methods like pointwise linear regression, the model can visually emphasize areas of significant worsening in the predicted VF using distinct colors. This enables clinicians to make timely decisions, such as rescheduling follow-ups or switching medications, when a decline is anticipated. Additionally, for patients whose results are predicted to remain stable for years, these visual projections can intuitively demonstrate the lack of significant change, thereby alleviating patient anxiety.

In conclusion, MDA-RNN outperformed our previously developed masked BiGRU model, and its two attention mechanisms were well-trained, consistent with previous VF studies. The MDA-RNN learned to focus more on important features such as TDV, PDV, MD, and FN in each input vector and selected more reliable VFs over all time steps. Thus, it could reduce the noise in the VF inputs and help recognize progression trends in VFs. Thus, with long prediction intervals, MDA-RNN is more robust in predicting more severe and largely worsening glaucoma.

## Methods

All training and internal test data were obtained from patients who visited glaucoma clinics at multi-center tertiary hospitals (Pusan National University Hospital, Kosin University Gospel Hospital, Dong-A University Hospital, Busan Paik Hospital, and Pusan National University Yangsan Hospital) between June 2004 and April 2022. The dataset was randomly split into training and test datasets at the patient level. This strict patient-level separation ensured that all longitudinal visual field examinations from a single patient were assigned exclusively to either the training or the test set, thereby strictly preventing any data leakage. This study was performed according to the tenets of the Declaration of Helsinki and approved by the Institutional Review Boards (IRB) of each hospital (Pusan National University Hospital (Approval No. 2203-018-113), Kosin University Gospel Hospital (Approval No. 2018-12-028), Dong-A University Hospital (Approval No. 22-074), Busan Paik Hospital (Approval No. 2023-11-179), and Pusan National University Yangsan Hospital (Approval No. 05-2018-172)). The requirement for patient consent was also waived by the IRB of each hospital because of the retrospective study design.

For the external test dataset, we utilized a large-scale public dataset released by Washington University.^32^ This dataset comprises 28,943 visual field examinations obtained from 7248 eyes of 3871 patients. Of the total 7248 eyes, only those with at least four visual field examinations were included in this study. As a result, only 18,683 visual field examinations from 2991 eyes were used in this study. As this dataset does not contain specific information regarding reliability indices (false negative ratio, false positive ratio, fixation loss ratio), these values were assigned a fixed value of 15% each for the purposes of this study. The value of 15% was chosen as a representative threshold for reliable visual field tests based on conventional clinical standards. The dataset did not include standard global indices. Specifically, MD was replaced by MTD (mean total deviation), which is the simple arithmetic mean of the TDVs. Similarly, the standard deviation of the TDVs was provided instead of PSD, and no VFI data was supplied. Therefore, we used the R package ‘visualFields’ (version 1.0.7) to estimate VFI values. For the Mean Deviation (MD) and Pattern Standard Deviation (PSD), we directly utilized the MTD and PSD values provided in the original dataset.

The training dataset consists of 154,105 VF exams from 22,404 eyes of 12,193 subjects; all subjects underwent at least 4 VF tests and mean follow-up duration was 1766 ± 1318 days (4.84 ± 3.61 years). The mean age was 57.1 ± 16.9 years old. The VF mean deviation (MD) and mean prediction time interval (the time interval between prediction and last VF examination) were − 7.81 ± 8.75 dB and 310.3 ± 334.6 days (0.85 ± 0.92 years), respectively (Table 1). We randomly split 154,105 training dataset records into training and validation datasets at the patient level in the ratio of 9:1. The validation data were used to check the current fitness of the neural network during training to prevent overfitting.

### Visual field examination

Automated perimetry was performed using a Humphrey visual field analyzer 750i instrument (Carl Zeiss Meditec, Inc., Dublin, CA, USA) with Swedish interactive threshold algorithms (SITA) 24-2 or 30-2. The 24-2 test pattern was selected for training or testing our model because it is widely established as the clinical standard for glaucoma diagnosis and routine practice^33^. When VF was measured using the 30-2 test pattern, 54 test points (including 2 points of physiologic blind spots) overlapping with the 24-2 test pattern were used. However, for these 30-2 visual fields, the original global indices were utilized instead of relying on approximate recalculations based on the 24-2 test locations. Glaucomatous VFs were those that met at least one of the following criteria: glaucoma hemifield test outside the normal limits and/or pattern standard deviation (PSD) probability outside of 95% of the normal population. The reliability criteria applied were fixation loss (FL) of less than 33%, false-positive (FP) rate of less than 33%, and false-negative (FN) rate of less than 33%.

### Deep learning architecture

We designed a MDA-RNN based on the existing DA-RNN^13^ architecture. The DA-RNN utilizes Bahdanau attention methods twice in the model, as encoder and decoder. In encoder attention, the MDA-RNN determines the important features of the input vector. Decoder attention determines which input vector is more important than others. The number of VF examinations per patient varies; however, the DA-RNN architecture requires a fixed number of input vectors. We modified the DA-RNN to support a masking mechanism, which ignores the empty input included in the fixed number of input vectors.

**Fig. 6:**
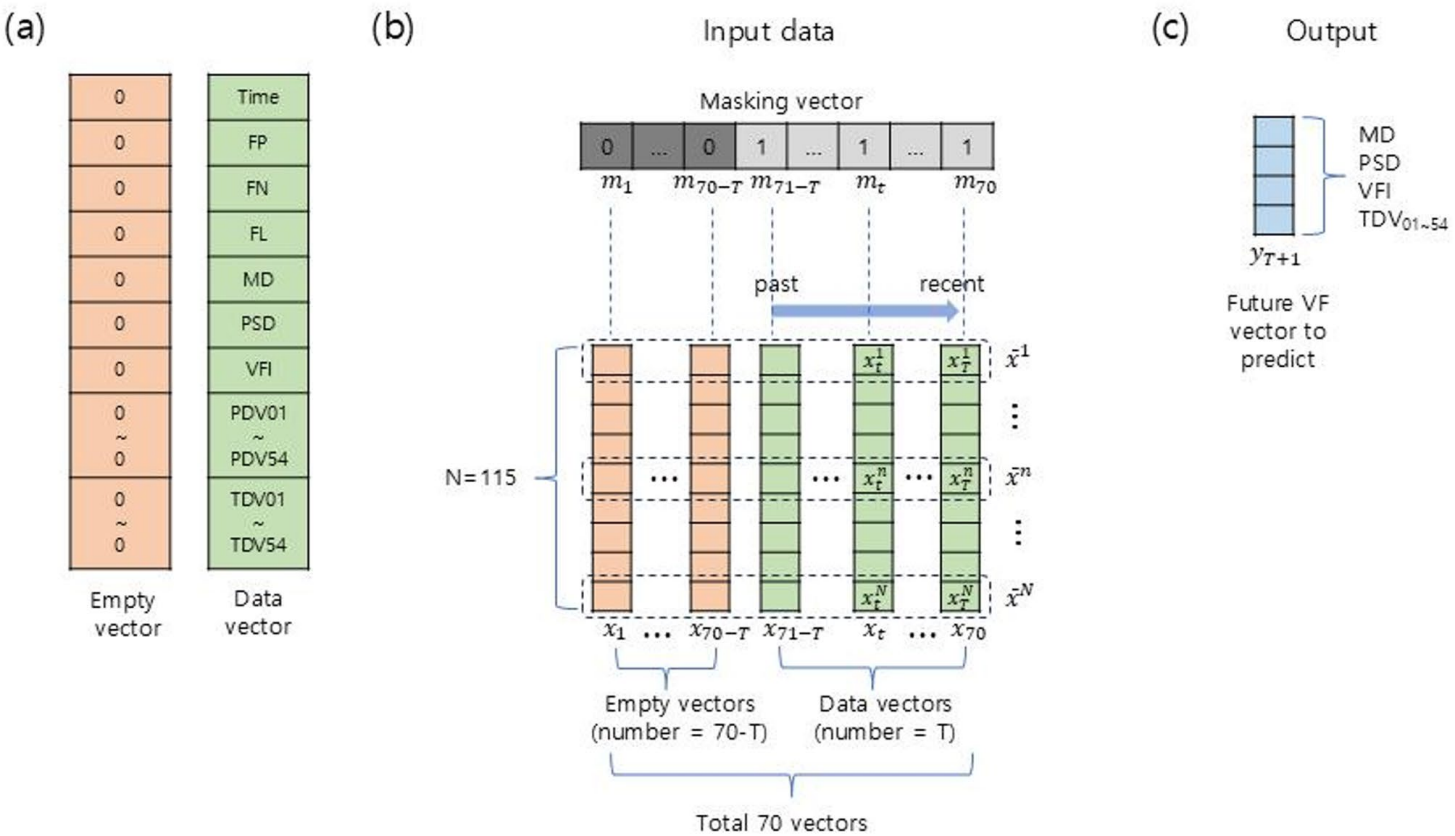
Input and output data structure and notations. Seventy input VF data consists of two kinds of vectors, empty and data vector. The number of data vector is varied per patient and denoted as T. Masking vector is another 1-dimensional input vector and its value is 0 (means empty vector) or 1 (means data vector). The output comprises 57 values of VF mean deviation (MD), pattern standard deviation (PSD), VF index (VFI), and 54 total deviation values (TDVs).

The input and output data formats are shown in Fig. 6. The input data consist of a bunch of 1-dimensional vectors of two types: empty and data vectors (Fig. 6a, b). Empty vectors are zero-filled vectors containing no information. The data vector contained actual VF data, such as MD, PSD, visual field index (VFI), 54 total deviation values (TDV), and 54 pattern deviation values (PDV). The total number of features contained in the data vector was 115 (time intervals between data vectors, FP, FN, FL, MD, PSD, VFI, 54 PDV values, and 54 TDV values). The input sequence length, which is the total number of input vectors, is set to 70 (empty vector + data vector = 70). The masking vector is another input vector consisting of 70 element values of 0 (means empty vector) or 1 (means data vector).

The notation used in our model is summarized in Fig. 6b. Input $X=({ x}_{1},\dots ,{ x}_{70-T},{ x}_{71-T},\dots , {x}_{t}, {\dots , x}_{70})$ and the transpose of X is $\overline{X }=\left({\overline{x} }^{1}, {\overline{x} }^{2}, \dots , {\overline{x} }^{N}\right)$ where ${x}_{1}-{ x}_{70-T}$ is a zero-filled empty vector (length = 115), ${x}_{71-T}-{ x}_{70}$ is the data vector (length = 115) at temporal sequence t, ${\overline{x} }^{n}$ is the n^th^ feature vector (length = 70), T is the total number of data vectors, and N (= 115) is the total number of features. Input mask vector is denoted as $M=({ m}_{1},\dots ,{ m}_{70-T},{ m}_{71-T}, {m}_{t}, {\dots , m}_{70}) =(\mathrm{0,0}\dots \mathrm{0,1},1,\dots 1)$.

**Fig. 7:**
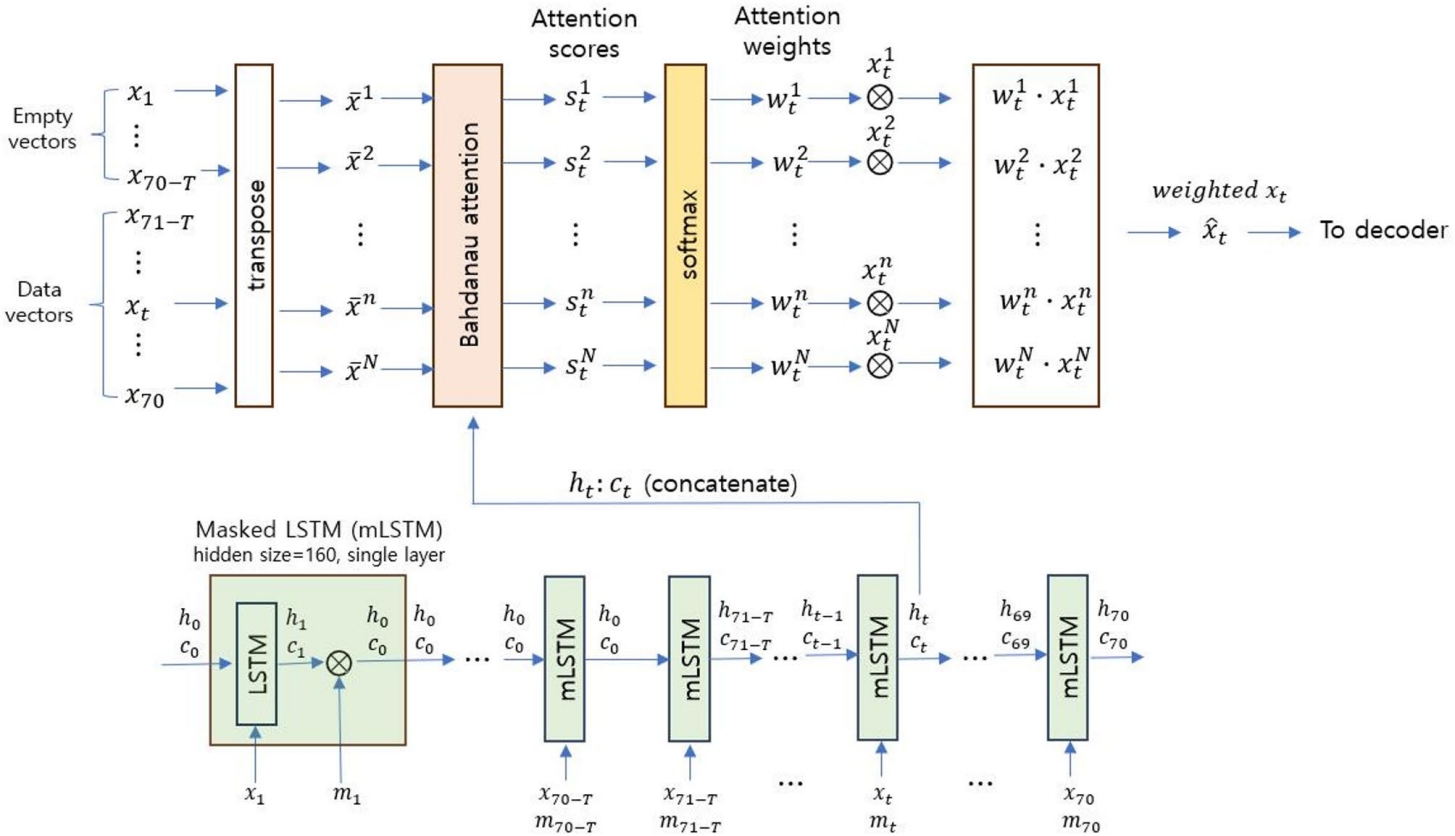
Encoder architecture. Masking operation was added after each long short term memory units (LSTM) operation. States of LSTM were multiplied with mask value and reset to 0 until last empty vector met.

The encoder structure and masking operations are described in Fig. 7. The overall structure is the same as that of the original DA-RNN but with the following differences: (1) there is a masking operation in LSTM called mLSTM. (2) Instead of using LSTM states at t-1, states at t ($${h}_{t}, {c}_{t}$$) were used in the Bahdanau attention operation. (3) Instead of using the LSTM hidden states as the encoder output, we used $$\widehat{x}$$. The encoder operation starts with input $$\left({x}_{t}, {m}_{t}\right)$$ to the LSTM and obtains the next hidden and cell states ($${h}_{t}, {c}_{t}$$). Then, ($${h}_{t}, {c}_{t}$$) is multiplied with the mask value $${m}_{t}$$, which means that until the data vector emerges, all ($${h}_{t}, {c}_{t}$$) are multiplied by 0 ($$={m}_{1}-{m}_{70-T})$$ and reset to zero states ($${h}_{0}, {c}_{0}).$$ In other words, the LSTM states were always reset to zero ($${h}_{0}, {c}_{0})$$ until a data vector emerged. ($${h}_{t}, {c}_{t}$$) were concatenated to $$\left[{h}_{t}:{c}_{t}\right]$$ and entered into the Bahdanau attention operation. Bahdanau attention score ($${s}_{t}^{n})$$ and weight ($${w}_{t}^{n})$$ were calculated as follows:

$$s_{t}^{n} = U_{3}^{T} \tanh \left( {U_{2} \left[ {h_{t} :c_{t} } \right] + U_{1} \bar{x}^{n} } \right)\;where\;U_{3} ,U_{2} ,\;and\;U_{1} \;are\;parameters\;to\;learn$$

$$w_{t}^{n} = Soft\max \left( {s_{t}^{n} } \right) = \frac{{\exp \left( {s_{t}^{n} } \right)}}{{\sum\nolimits_{{i = 1}}^{N} {\exp } \left( {s_{t}^{i} } \right)}}a$$

After calculating all attention weights, the attention weight vector ${{w}_{t}=(w}_{t}^{1},{w}_{t}^{2},\dots ,{w}_{t}^{n},{\dots , w}_{t}^{N})$ is obtained. Finally, the weight vector is multiplied element-wise by the input vector ${x}_{t}$ to obtain the weighted input ${\widehat{x}}_{t}$ as follows:

$$\hat{x}_{t} = w_{t} *x_{t} = \left( {w_{t}^{1} \cdot 0,w_{t}^{2} \cdot 0,w_{t}^{n} \cdot 0,w_{t}^{N} \cdot 0} \right) = \left( {0,0, \ldots 0,0} \right)\;for\;empty\;vectors\;(t = 1\;to\;70 - T)$$

$$\hat{x}_{t} = w_{t} *x_{t} = \left( {w_{t}^{1} \cdot x_{t}^{1} ,w_{t}^{2} \cdot x_{t}^{2} , \ldots ,w_{t}^{n} \cdot x_{t}^{n} , \ldots ,w_{t}^{N} \cdot x_{t}^{N} } \right)for\;data\;vectors\;(t = 71 - T\;to\;70).$$

Then, the LSTM moves to the next input vector and repeats the above operation until all input vectors are processed. Finally, the encoder output is $\hat{X} = \left( {\hat{x}_{1} , \ldots , \hat{x}_{70} } \right)$.

**Fig. 8:**
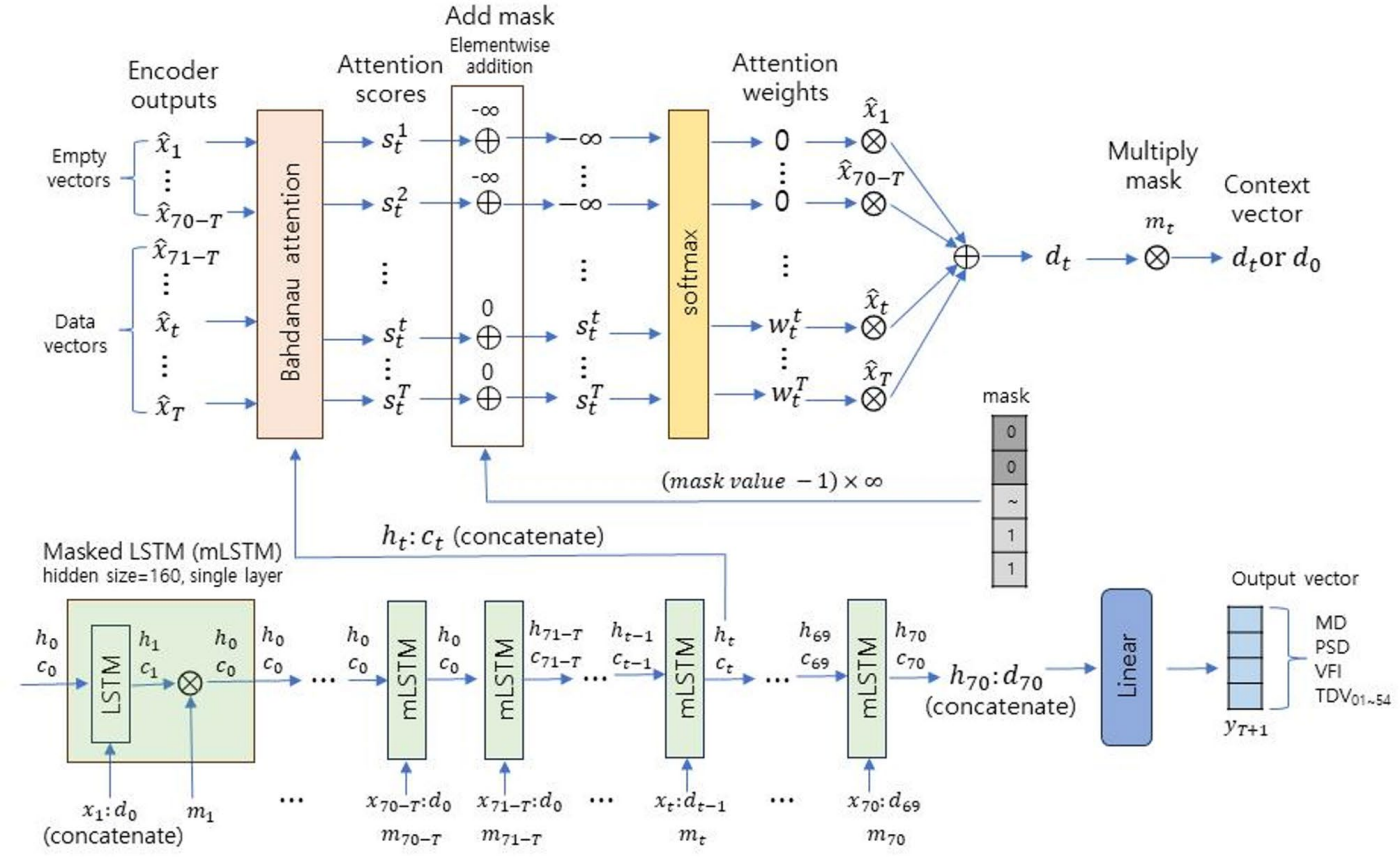
Decoder architecture. The same masking operation was also used after long short term memory units (LSTM). Mask values were also added to attention scores to make weights of empty vectors zero.

The decoder structure is described in Fig. 8. The overall structure is the same as that of the original DA-RNN but with the following differences: (1) the decoder takes three types of input data: encoder output ($$\widehat{X}$$), mask vector M, and VF input X (the same as the encoder VF input). (2) There is a mask operation in the attention and LSTM operations. Each VF input vector $${x}_{t}$$ and context vector $${d}_{t-1}$$ were concatenated to [$${x}_{t}:{d}_{t-1}]$$ before entering the mLSTM. Subsequently, the same mask operation as the encoder that resets the LSTM states to zero until the data vector emerges (t = 1 to 70-T) was performed. After the mask operation, the hidden and cell states of the mLSTM were concatenated to $$\left[{h}_{t}:{c}_{t}\right]$$ and entered into the Bahdanau attention operation. The Bahdanau attention score ($${s}_{t}^{i})$$ was calculated as follows:

$$s_{t}^{i} = U_{3}^{T} \tanh \left( {U_{2} \left[ {h_{t} :d_{{t - 1}} } \right] + U_{1} \hat{x}_{t} } \right)\;where\;U_{3} ,U_{2} ,U_{1} are\;the\;parameters\;to\;learn$$.

Mask operation to attention scores starts with mask conversion = $\left(mask value-1\right)\times \infty$ which converts (0, 1) values in the mask vector to $(-\infty , 0)$ and then adds element-wise to the decoder attention scores (${s}_{t}^{1}\dots {s}_{t}^{70}$), which transforms all empty vector scores to $-\infty$ and leave intact data vector values. The softmax function converts the scores to weights, where $-\infty$ scores are converted to zero and the other scores are converted to weight values. Each weight value is multiplied by the encoder output vectors, and the weighted vectors are then added to obtain the context vector ${d}_{t}$. The mask value ${m}_{t}$ is multiplied by ${d}_{t}$ to reset the context vector to ${d}_{0}$ until the LSTM meets the data vectors. After the LSTM scans all the input vectors, the final hidden state ${h}_{70}$ is concatenated with ${d}_{70}$ $[{h}_{70}:{d}_{70}]$ and enters the following linear layer to generate the final output vector.

### Deep learning model training

An adaptive moment estimation optimizer (Adam) (learning rate = 0.001) was used to train the MDA-RNN model and mean square error (MSE) was used as its loss function. The batch size was 16 and an early stopping function with a patience of 20 was used to stop the training automatically. The MDA-RNN model was developed using Python 3.12 libraries, including PyTorch 2.2.2, CUDA version, NumPy 1.26.3, and SciPy 1.13.1. Our previous model, masked BiGRU, was also retrained because the training and test datasets were changed. The masked BiGRU model was trained in the same environment as previously used. An Adam optimizer (learning rate = 0.001) was used, and MSE was used for its loss function. The batch size was 20, and an early stopping function with a patience of 20 was used to automatically stop the training. The masked BiGRU model was developed using Python 3.10 libraries, including TensorFlow 2.12.0, Keras 2.12.0, NumPy 1.23.5, and SciPy 1.10.1. The python source code has been made available as supplementary material.

### Statistical analyses

The pointwise mean absolute error (MAE) and root mean square error (RMSE) of the TDV (TDV_P-MAE_ and TDV_P-RMSE_, respectively) were used to compare the prediction performance as follows:

$$pointwise\;MAE\;of\;TDV = TDV_{{P - MAE}} = \frac{1}{{54}}\sum\limits_{{n = 1}}^{{54}} {\left| {trueTDV_{n} - predictedTDV_{n} } \right|} ,$$

$$pointwise\;RMSE\;of\;TDV = TDV_{{P - RMSE}} = \sqrt {\frac{1}{{54}} \times \sum\limits_{{n = 1}}^{{54}} {\left( {trueTDV_{n} - predictedTDV_{n} } \right)^{2} } } ,$$

For the global indices, MD, PSD, and VFI, the absolute error between the true and predicted values was calculated for each eye. The Shapiro–Wilk test was performed to check the normality of the data distribution and nonparametric paired tests, and Wilcoxon’s signed rank test was used to compare the performance between the new MDA-RNN model and the previous masked BiGRU model. To account for the increased risk of Type I errors arising from multiple comparisons in our subgroup analyses, we applied the Benjamini–Hochberg procedure to control the false discovery rate (FDR). Statistical significance for these analyses was determined using the FDR-adjusted *p* values, with an adjusted *p* value of < 0.05 considered statistically significant. Statistical analyses were performed using R statistics (version 4.0.3 for Windows). Matplotlib 3.9.0, Seaborn 0.13.2, and scikit-learn 1.5.0 were also used to draw plots. To perform a broken-stick (piecewise linear regression) analysis between the signed prediction error and changes in mean total deviation value (MTD). MTD is arithmetic mean of the 54 total deviation values defined as follows:

$$mean\;total\;deviation,MTD = \frac{1}{{54}} \times \sum\limits_{{n = 1}}^{{54}} {TDV_{n} }$$

Signed prediction error is MTD_true_–MTD_predicted_ and △MTD (= MTD_true_–MTD_last-observed_) is the difference between the last (most recent) observed MTD and the true MTD at the time of prediction. A python library piecewise-regression^34^ 1.5.0 was used.

## Supplementary Information

Below is the link to the electronic supplementary material.

Supplementary Material 1

## Data Availability

The data supporting the findings of this study are available from the corresponding author (JWL) upon reasonable request. Python source codes used in this study are provided in the supplementary files.

## References

[1] Resnikoff, S. et al. Global data on visual impairment in the year 2002. *Bull. World Health Organ.* **82**, 844–851 (2004). 15640920 PMC2623053

[2] Weinreb, R. N., Aung, T. & Medeiros, F. A. The pathophysiology and treatment of glaucoma: A review. *JAMA* **311**, 1901–1911 (2014). 24825645 10.1001/jama.2014.3192 PMC4523637

[3] Aref, A. A. & Budenz, D. L. Detecting visual field progression. *Ophthalmology* **124**, S51–S56 (2017). 29157362 10.1016/j.ophtha.2017.05.010

[4] Wilkins, M. R., Fitzke, F. W. & Khaw, P. T. Pointwise linear progression criteria and the detection of visual field change in a glaucoma trial. *Eye* **20**, 98–106 (2006). 15650759 10.1038/sj.eye.6701781

[5] Murata, H., Araie, M. & Asaoka, R. A new approach to measure visual field progression in glaucoma patients using variational Bayes linear regression. *Invest. Ophthalmol. Vis. Sci.* **55**, 8386–8392 (2014). 25414192 10.1167/iovs.14-14625

[6] Dixit, A., Yohannan, J. & Boland, M. V. Assessing glaucoma progression using machine learning trained on longitudinal visual field and clinical data. *Ophthalmology* **128**, 1016–1026 (2021). 33359887 10.1016/j.ophtha.2020.12.020 PMC8222148

[7] Wen, J. C. et al. Forecasting future Humphrey visual fields using deep learning. *PLoS ONE* **14**, e0214875 (2019). 30951547 10.1371/journal.pone.0214875 PMC6450620

[8] Moradi, M. et al. PyGlaucoMetrics: A stacked weight-based machine learning approach for glaucoma detection using visual field data. *Medicina* **61**, 541 (2025). 40142352 10.3390/medicina61030541 PMC11944261

[9] Park, K., Kim, J. & Lee, J. Visual field prediction using recurrent neural network. *Sci. Rep.* **9**, 8385 (2019). 31182763 10.1038/s41598-019-44852-6 PMC6557823

[10] Kim, H. et al. Visual field prediction using a deep bidirectional gated recurrent unit network model. *Sci. Rep.* **13**, 11154 (2023). 37429862 10.1038/s41598-023-37360-1 PMC10333213

[11] Lee, J. et al. Bidirectional gated recurrent unit network model can generate future visual field with variable number of input elements. *PLoS ONE* **19**, e0307498 (2024). 39190660 10.1371/journal.pone.0307498 PMC11349096

[12] Vaswani, A. et al. Attention is all you need. *Adv. Neural Inf. Process. Syst.* **30**, 1 (2017).

[13] Qin, Y. *et al.* A dual-stage attention-based recurrent neural network for time series prediction. Preprint at http://arxiv.org/abs/1704.02971 (2017).

[14] Heijl, A., Lindgren, A. & Lindgren, G. Test-retest variability in glaucomatous visual fields. *Am. J. Ophthalmol.* **108**, 130–135 (1989). 2757094 10.1016/0002-9394(89)90006-8

[15] Hutchings, N., Wild, J. M., Hussey, M. K., Flanagan, J. G. & Trope, G. E. The long-term fluctuation of the visual field in stable glaucoma. *Invest. Ophthalmol. Vis. Sci.* **41**, 3429–3436 (2000). 11006235

[16] Boeglin, R. J., Caprioli, J. & Zulauf, M. Long-term fluctuation of the visual field in glaucoma. *Am. J. Ophthalmol.* **113**, 396–400 (1992). 1558113 10.1016/s0002-9394(14)76161-6

[17] Blumenthal, E. Z. et al. Comparison of long-term variability for standard and short-wavelength automated perimetry in stable glaucoma patients. *Am. J. Ophthalmol.* **129**, 309–313 (2000). 10704545 10.1016/s0002-9394(99)00432-8

[18] Rabiolo, A. et al. Comparison of methods to detect and measure glaucomatous visual field progression. *Trans. Vis. Sci. Tech.* **8**, 2 (2019). 10.1167/tvst.8.5.2 PMC6748341 31555493

[19] Artes, P. H., Nicolela, M. T., LeBlanc, R. P. & Chauhan, B. C. Visual field progression in glaucoma: Total versus pattern deviation analyses. *Invest. Ophthalmol. Vis. Sci.* **46**, 4600–4606 (2005). 16303955 10.1167/iovs.05-0827

[20] Yohannan, J. et al. Evidence-based criteria for assessment of visual field reliability. *Ophthalmology* **124**, 1612–1620 (2017). 28676280 10.1016/j.ophtha.2017.04.035 PMC5675138

[21] Rao, H. L. et al. Role of visual field reliability indices in ruling out glaucoma. *JAMA Ophthalmol.* **133**, 40–44 (2015). 25256758 10.1001/jamaophthalmol.2014.3609

[22] Tan, N. Y. Q. et al. The effect of testing reliability on visual field sensitivity in normal eyes: The Singapore Chinese Eye Study. *Ophthalmology* **125**, 15–21 (2018). 28863943 10.1016/j.ophtha.2017.08.002

[23] Pascual, J. P. et al. Spatial characteristics of visual field progression determined by Monte Carlo simulation: Diagnostic innovations in glaucoma study. *Invest. Ophthalmol. Vis. Sci.* **48**, 1642–1650 (2007). 17389495 10.1167/iovs.06-0966

[24] Boden, C. et al. Patterns of glaucomatous visual field progression identified by three progression criteria. *Am. J. Ophthalmol.* **138**, 1029–1036 (2004). 15629296 10.1016/j.ajo.2004.07.003

[25] Mikelberg, F. S. & Drance, S. M. The mode of progression of visual field defects in glaucoma. *Am. J. Ophthalmol.* **98**, 443–445 (1984). 6486216 10.1016/0002-9394(84)90128-4

[26] Birt, C. M. et al. Analysis of reliability indices from Humphrey visual field tests in an urban glaucoma population. *Ophthalmology* **104**, 1126–1130 (1997). 9224465 10.1016/s0161-6420(97)30173-0

[27] McMillan, T. A., Stewart, W. C. & Hunt, H. H. Association of reliability with reproducibility of the glaucomatous visual field. *Acta Ophthalmol.* **70**, 665–670 (1992). 1471493 10.1111/j.1755-3768.1992.tb02150.x

[28] Rabiolo, A. et al. Predictors of long-term visual field fluctuation in glaucoma patients. *Ophthalmology* **127**, 739–747 (2020). 31952885 10.1016/j.ophtha.2019.11.021

[29] de Moraes, C. G., Furlanetto, R. L., Ritch, R. & Liebmann, J. M. A new index to monitor central visual field progression in Glaucoma. *Ophthalmology* **121**, 1531–1538 (2014). 24726202 10.1016/j.ophtha.2014.02.007

[30] Eslami, M. et al. Visual field prediction: Evaluating the clinical relevance of deep learning models. *Ophthalmol. Sci.* **3**, 100222 (2023). 36325476 10.1016/j.xops.2022.100222 PMC9619031

[31] Saunders, L. J., Medeiros, F. A., Weinreb, R. N. & Zangwill, L. M. What rates of glaucoma progression are clinically significant?. *Expert Rev. Ophthalmol.* **11**, 227–234 (2016). 29657575 10.1080/17469899.2016.1180246 PMC5898440

[32] Montesano, G., Chen, A., Lu, R., Lee, C. S. & Lee, A. Y. UWHVF: A real-world, open source dataset of perimetry tests from the humphrey field analyzer at the University of Washington. *Trans. Vis. Sci. Tech.* **11**, 2 (2022). 10.1167/tvst.11.1.1 PMC8742531 34978561

[33] Onyekaba, N.-A.E. et al. Comparison of 10-2 and 24-2 perimetry to diagnose glaucoma using OCT as an independent reference standard. *Ophthalmol. Glaucoma* **6**, 187–197 (2023). 36084839 10.1016/j.ogla.2022.08.017 PMC10281760

[34] Pilgrim, C. Chasmani/piecewise-regression. (2024).

