# Supplementary material for: Predicting worsening visual field via masked dual stage attention recurrent neural network
Source: Sci Rep. 2026 Apr 30;16:20192. doi: 10.1038/s41598-026-51152-3 (PMC13324851; doi:10.1038/s41598-026-51152-3)
Supplement: Supplementary file 1 — Supplementary Material 1 [file 41598_2026_51152_MOESM1_ESM.pdf]

```python
import data_reader
import tqdm
import numpy as np
import torch
from torch.utils.data import DataLoader
from MDARNN import get_model

# ================================================================================
model_filepath = "./models/DARNN_final_model.pth"
excel_filepath = "./data/test_data.xlsm"  # VF excel file
batch_size = 64
# ================================================================================

x_test, y_test, demo = data_reader.open_excel_file(excel_filepath,
                                                   check_reliability=True, max_row=0,
                                                   relative_exam_interval=True,
                                                   date_ascending=True, empty_row_last=False)

print("Loading completed. Total test set: {}".format(len(x_test)))

last_x = np.array(x_test)[:, -1, :]

x_enc = np.array(x_test, dtype=float)
y = np.array(y_test, dtype=float)

test_dataset = data_reader.VFDataset(x_enc, y, phase='test')
test_dataloader = DataLoader(test_dataset, batch_size=batch_size, shuffle=False)

device = torch.device("cuda:0" if torch.cuda.is_available() else "cpu")
model = get_model()
model.load_state_dict(torch.load(model_filepath))
model = model.to(device)
model.eval()

print("₩nTesting started. Device: {}".format(device))
with torch.no_grad():
    y_pred = np.empty((0, 57))
    ae = np.empty((0, 57))
    for x1, y1 in tqdm.tqdm(test_dataloader):
        x1 = x1.to(device)
        outputs = model(x1)
        outputs = outputs.cpu().numpy()
        y1 = y1.cpu().numpy()
        ae = np.append(ae, np.abs(outputs - y1), axis=0)
        y_pred = np.append(y_pred, outputs, axis=0)

gl_mae = {'avg': np.average(ae[:, :3], axis=0), 'std': np.std(ae[:, :3], axis=0)}
tdv_mae = {'avg': np.average(np.average(ae[:, 3:], axis=1), axis=0),
           'std': np.std(np.average(ae[:, 3:], axis=1), axis=0)}
tdv_rmse = {'avg': np.average(np.sqrt(np.average(np.square(ae[:, 3:]), axis=1)), axis=0),
            'std': np.std(np.sqrt(np.average(np.square(ae[:, 3:]), axis=1)), axis=0)}

print("MDA-RNN performance")
print("====================================================")
print(f"No. of eyes: {len(y_test)}")
print(f"MD {gl_mae['avg'][0]:.2f} ± {gl_mae['std'][0]:.2f}, PSD {gl_mae['avg'][1]:.2f} ± {gl_mae['std'][1]:.2f}, VFI {gl_mae['avg'][2]:.2f} ± {gl_mae['std'][2]:.2f}")
```

```
print(f"TDV MAE {tdv_mae['avg']:.2f} ± {tdv_mae['std']:.2f}, TDV RMSE {
tdv_rmse['avg']:.2f} ± {tdv_rmse['std']:.2f}")
```

```python
import random
import data_reader
import numpy as np
import os
import tqdm
import time
import torch
from torch.utils.data import DataLoader
from MDARNN import get_model

# ==============================================================================
model_save_folder = ".₩₩models"  # folder to save weight file
excel_filepath = ".₩₩data₩₩train_data.xlsm"  # VF data file
pickle_folder = ".₩₩data"
demo_output_folder = "Z:₩₩MDA-RNN₩₩stat"  # folder to save patients' demographic data.
load_from_pickle = False  # To read data from VF excel file, False. (slow)


batch_size = 16
val_ratio = 0.1
shuffle = True
num_epochs = 200
auto_stop_tolerance = 20
# ==============================================================================


if load_from_pickle:
    x_all, y_all, demo = data_reader.open_pickle(pickle_folder, 'train_data')
else:
    x_all, y_all, demo = data_reader.open_excel_file(excel_filepath,
                                                     check_reliability=True, max_row=0,
                                                     relative_exam_interval=True,
                                                     date_ascending=True, empty_row_last=False)
    data_reader.save_pickle(x_all, y_all, demo, pickle_folder, 'train_data')

val_cnt = int(len(x_all)*val_ratio)
print("Loading completed. Total data set: {}, val: {}, train {}".format(len(x_all), val_cnt, len(x_all)-val_cnt ))

if demo_output_folder is not None:
    data_reader.save_as_csv(demo, demo_output_folder + "₩₩train_ids.csv")

if shuffle:
    idx = [i for i in range(len(x_all))]
    random.shuffle(idx)
    x_all = [x_all[i] for i in idx]
    y_all = [y_all[i] for i in idx]

x_enc = np.array(x_all, dtype=float)
y = np.array(y_all, dtype=float)

val_dataset = data_reader.VFDataset(x_enc[:val_cnt], y[:val_cnt], phase='val')
train_dataset = data_reader.VFDataset(x_enc[val_cnt:], y[val_cnt:], phase='train')
val_dataloader = DataLoader(val_dataset, batch_size=batch_size, shuffle=False)
train_dataloader = DataLoader(train_dataset, batch_size=batch_size, shuffle=False)
dataloader_dict = {'train': train_dataloader, 'val': val_dataloader}

# Initialize model
```

```
print("==> Initializing DA-RNN model ...")
device = torch.device("cuda:0" if torch.cuda.is_available() else "cpu")
model = get_model().to(device)
# summary(model, input_size=(batch_size, data_reader.timestep_size, 115))
# optimizer = torch.optim.SGD(model.parameters(), lr=0.001, momentum=0.9)
optimizer = torch.optim.Adam(model.parameters(), lr=0.001)
criterion = torch.nn.MSELoss().to(device)

# Train
print("==> Start training ...")
# ====================== checkpoint recovery ==========================
start_epoch = 0
best_loss = float("inf")
tolerance_cnt = 0
last_chkpt_file = ""
for f in os.listdir(model_save_folder):
    if f.split('_')[0] == "Checkpoint" and os.path.splitext(f)[1] == ".tar":
        checkpoint = torch.load(os.path.join(model_save_folder, f))
        start_epoch = checkpoint['epoch'] + 1
        best_loss = checkpoint['best_loss']
        model.load_state_dict(checkpoint['model_state_dict'])
        optimizer.load_state_dict(checkpoint['optim_state_dict'])
        last_chkpt_file = f
        break

# ========================== train begin ==============================
time_start = time.time()
print("₩nTraining started. Device: {}".format(device))
for epoch in range(start_epoch, num_epochs):
    print('₩n[Epoch {}/{}]   best-loss: {:.5f}'.format(epoch+1, num_epochs, best_loss))
    print('-'*60)
    for phase in ['train', 'val']:
        if phase == 'train':
            model.train()
        else:
            model.eval()

        # ======== epoch begin ==========
        epoch_loss = 0.0
        mae = np.array([0.0 for i in range(57)])
        for x, y in tqdm.tqdm(dataloader_dict[phase]):
            x = x.to(device)
            y = y.to(device)
            optimizer.zero_grad()
            with torch.set_grad_enabled(phase == 'train'):
                outputs = model(x)
                loss = criterion(outputs, y)
                if phase == 'train':
                    loss.backward()
                    optimizer.step()
                if phase == 'val':
                    output2 = outputs.cpu().numpy()
                    y2 = y.cpu().numpy()
                    mae += np.sum(np.abs(output2 - y2), axis=0)
                epoch_loss += loss.item() * x.size(0)
        # ======== epoch end ==========

        epoch_loss /= len(dataloader_dict[phase].dataset)
        if phase == 'train':
            print('{} Loss: {:.5f}'.format(phase, epoch_loss))
        if phase == 'val':
```

```
            mae /= len(dataloader_dict[phase].dataset)
            tdv_mae = np.sum(mae[3:], axis=0) / 54.0
            print('{} Loss: {:.5f} MAEs: MD {:.4f}, PSD {:.4f}, VFI {:.4f}, TDV {:.4f}'.format(phase, epoch_loss, mae[0], mae[1], mae[2], tdv_mae))
            if epoch_loss < best_loss:
                best_loss = epoch_loss
                best_model_wts = model.state_dict()
                model_filename = "Epoch_{:04d}_Loss_{:.5f}.pth".format(epoch+1, epoch_loss)
                torch.save(best_model_wts, os.path.join(base_folder + model_save_folder, model_filename))
                print(">> model saved. {}".format(model_filename))
                tolerance_cnt = 0
            else:
                tolerance_cnt += 1
                print("Tolerance count increased to {}/{}".format(tolerance_cnt, auto_stop_tolerance))

    # save checkpoint
    if last_chkpt_file != "":
        os.remove(os.path.join(base_folder + model_save_folder, last_chkpt_file))
    last_chkpt_file = "Checkpoint_Epoch_{:d}_Loss_{:.4f}.tar".format(epoch + 1, epoch_loss)
    torch.save({
                'epoch': epoch,
                'best_loss': best_loss,
                'model_state_dict': model.state_dict(),
                'optim_state_dict': optimizer.state_dict()}
               , os.path.join(base_folder + model_save_folder, last_chkpt_file))

    # auto-stop
    if tolerance_cnt >= auto_stop_tolerance:
        print("Maximum tolerance reached. Training finished.")
        break

time_elapsed = time.time() - time_start
print('Training completed in {:.0f}m {:.0f}s'.format(time_elapsed // 60, time_elapsed % 60))
print('Best loss: {:.5f}'.format(best_loss))
```

```python
import torch
import numpy as np
from torch import nn
import data_reader

# ======================================================
# T: time series length
# n: input data feature length
# m: encoder/decoder lstm features
# p: decoder lstm features
# target_len : final output length

# encoder input data shape = (batch size, T, n)
# decoder input data shape = (batch size, T, n)
# ======================================================


def get_model():
    return DARNN(T=data_reader.timestep_size, n=115, m=160, p=160, target_len=57)


class MaskedBahdanauAttention(nn.Module):
    def __init__(self, query_size, key_size, filter_size=None):
        """
        :param query_size:
        :param key_size:
        :param filter_size: 내부 연산 행렬 크기. None 일경우 key_size로 세팅
        """
        super(MaskedBahdanauAttention, self).__init__()
        if filter_size is None:
            filter_size = key_size
        self.w1 = nn.Linear(key_size, filter_size)  # for key
        self.w2 = nn.Linear(query_size, filter_size)  # for query
        self.w3 = nn.Linear(filter_size, 1)  # for value

    def forward(self, query, key, mask=None):
        """
        :param query: shqpe=(batch, query_size)
        :param key: shape=(batch, seq_length, key_size)
        :param mask: shape=(batch, seq_length) for key. 0=invalid, 1=valid
        :return: shape=(batch, seq_length)
        """
        query = query.unsqueeze(dim=1).repeat(1, key.size()[1], 1)
        score = torch.tanh(self.w1(key) + self.w2(query))
        score = self.w3(score).squeeze(dim=2)  # batch, T, 1
        if mask is not None:
            # 무효벡터를 음의 무한대로 전환해 softmax 가중치가 0이 되도록 한다.
            mask_conv = 10000000000 * (mask - 1)
            score = torch.add(score, mask_conv)
        attn_weights = torch.softmax(score, dim=1)  # 각 key 의 중요도를 의미
        return attn_weights, score


class Encoder(nn.Module):
    def __init__(self, T, n, m):
        super(Encoder, self).__init__()
        """
        T : timestep length
        n : 입력데이터의 number of features
        m : 인코더 LSTM의 Units(outputs) 개수. (lstm의 출력을 몇개의 차원으로 할 것인가?)
        """
        self.m = m
```

```python
        self.input_attn = MaskedBahdanauAttention(key_size=T, query_size=2 * m)
        self.lstm = nn.LSTM(input_size=n, hidden_size=m, num_layers=1, batch_first=True)

    def forward(self, data):
        """
        data : encoder data (shape = batch, T, n)
        """
        batch_size = data.size()[0]
        timestep_size = data.size()[1]
        n = data.size()[2]

        mask = torch.sum(torch.abs(data), dim=2)
        mask = torch.where(mask > 0, 1.0, 0)  # batch, T

        # reset state : shape = (num_layer, batch, hidden_size)
        h_s = torch.zeros((self.lstm.num_layers, batch_size, self.lstm.hidden_size), device=data.device)
        c_s = torch.zeros((self.lstm.num_layers, batch_size, self.lstm.hidden_size), device=data.device)

        alpha_stack = torch.empty((batch_size, 0, n), dtype=torch.float32, device=data.device)
        key_permuted = torch.permute(data, (0, 2, 1))  # batch, n, T
        for t in range(timestep_size):
            # 배치단위로 각 시간순서의 값들을 떼어서 3차원으로 변환시켜준다
            x = data[:, t, :].unsqueeze(dim=1)  # x : (batch, 1, n)

            # 각 배치의 모든 시 1 time을 넣어서 lstm을 수행한다. (1 time step lstm)
            _, (h_s, c_s) = self.lstm(x, (h_s, c_s))  # h_s, c_s : shape 주의! 항상 (num_layer, batch, m) 임.

            # 무효 입력이 들어온 경우 출력을 다 초기화 시킨다.
            h_s = torch.multiply(h_s, mask[:, t].unsqueeze(dim=0).unsqueeze(dim=2))
            c_s = torch.multiply(c_s, mask[:, t].unsqueeze(dim=0).unsqueeze(dim=2))

            # 1 time step에 대한 input attetnion을 진행한다. h_s, c_s 에서 num_layer 차원을 버리고 넘겨준다.
            # 논문에서 hidden과 cell state를 concat하여 어텐션하는게 정확도가 더 높다고 한다.
            query = torch.cat((h_s.squeeze(dim=0), c_s.squeeze(dim=0)), dim=1)
            alpha_t, _ = self.input_attn(query, key_permuted)  # batch, n
            alpha_t = alpha_t.unsqueeze(dim=1)  # batch, 1, n
            # 한 시계열에 대한 feature attention weight가 출력된다.

            # 이후에 곱을 한꺼번에 진행하기 위해서 value와 곱하는 과정을 먼저 수행하진 않고 차곡차곡 쌓아둔다.
            # alpha_stack 의 최종 shape: batch, T, n
            alpha_stack = torch.cat((alpha_stack, alpha_t), dim=1)


        # Attention Weight * Value를 통해, 실제 Context_Vector set를 구하게 된다.
        # 결과적으로는 각 시간 time-line별로 가장 중요하게 생각되는 특성들만 값이 높아지게 된다.
        x_carrot = torch.multiply(data, alpha_stack)  # batch, T, n
        return x_carrot, alpha_stack  # batch, T, n


class Decoder(nn.Module):
    def __init__(self, p, m, n_dec):
```

```python
        super(Decoder, self).__init__()
        self.m = m
        self.p = p
        self.temp_attn = MaskedBahdanauAttention(query_size=2*p, key_size=m)
        self.lstm = nn.LSTM(input_size=m+n_dec, hidden_size=p, num_layers=1, batch_first=True)

    def forward(self, data, enc_output, mask_enc):
        """
        data : decoder input data (shape = batch, T, n)
        enc_output : encoder output (shape = batch, T, n)
        mask_enc : batch, T
        """
        batch_size = data.size()[0]
        timestep_len = data.size()[1]

        mask_dec = torch.sum(torch.abs(data), dim=2)
        mask_dec = torch.where(mask_dec > 0, 1.0, 0)

        # reset state : shape = num_layer, batch, hidden_size
        h_s = torch.zeros((self.lstm.num_layers, batch_size, self.lstm.hidden_size), device=data.device)
        c_s = torch.zeros((self.lstm.num_layers, batch_size, self.lstm.hidden_size), device=data.device)

        # context_v 초기화
        context_v = torch.zeros((batch_size, 1, self.m), device=data.device)  # batch,1,m
        beta_sum = None
        score = None
        for t in range(timestep_len):
            # decoder에 집어넣은 data를 모든 배치별 1 time step으로 분리한다.
            x = data[:, t, :]  # (batch, n)
            x_cat = torch.concat((x.unsqueeze(dim=1), context_v), dim=2)

            # attention을 진행할 decoder의 hidden과 cell을 구한다.
            _, (h_s, c_s) = self.lstm(x_cat, (h_s, c_s))  # h_s, c_s 차원 주의: num_layer, batch, p
            h_s = torch.multiply(h_s, mask_dec[:, t].unsqueeze(dim=0).unsqueeze(dim=2))
            c_s = torch.multiply(c_s, mask_dec[:, t].unsqueeze(dim=0).unsqueeze(dim=2))

            # encoding에서 weighted sum된 특징값들을 통해서 (특징 중요도로 학습한 결과로), decoder에서는 시간에 대한 attention이 진행되게 된다.
            # 결과는 시간들에 있어서 가장 중요한 값이 뭐였는지에 대한 weight가 나온다
            query = torch.cat((h_s.squeeze(dim=0), c_s.squeeze(dim=0)), dim=1)
            beta_t, score = self.temp_attn(query, enc_output, mask_enc)  # batch, T
            beta_t = beta_t.unsqueeze(dim=2)  # batch, T, 1
            beta_t = torch.permute(beta_t, (0, 2, 1))  # batch, 1, T
            # 실제로 encoder 시간값들(사실상 value)에 곱해서 가장 유의미했던 시간값들의 특징값에는 더 많은 가중치가 곱해지게 된다.
            context_v = torch.matmul(beta_t, enc_output)  # batch,1,m

            # 무효 입력이 들어온 경우 출력을 다 초기화 시킨다.
            context_v = torch.multiply(context_v, mask_dec[:, t].unsqueeze(dim=1).unsqueeze(dim=2))

            beta_t = beta_t.squeeze(dim=1)
            beta_t = torch.multiply(beta_t, mask_dec[:, t].unsqueeze(dim=1))
            beta_sum = beta_t if beta_sum is None else torch.add(beta_sum, beta_t)
```

```
        # h_s 의 num_layer 차원을 없앤 후 가운데 1차원을 추가 하여 batch, 1, p 를 만든다.
        dec_h = h_s.squeeze(dim=0).unsqueeze(dim=1)

        # context_v 와 합쳐서 출력을 만든다.
        output = torch.cat((dec_h, context_v), dim=2)  # batch, 1, m+p
        return output, beta_sum.squeeze(dim=1), score.squeeze(dim=1)  # (batch, 1, m+p), (batch, T)


class DecoderTail(nn.Module):
    def __init__(self, in_features, out_features):
        super(DecoderTail, self).__init__()
        self.net = nn.Sequential(
            nn.Linear(in_features, out_features)
        )

    def forward(self, data):
        out = self.net(data)
        out = norm_y(out)
        return out


class DARNN(nn.Module):
    def __init__(self, T, n, m, p, target_len):
        super(DARNN, self).__init__()
        """
        T : time step size
        n : input feature length
        m : encoder lstm feature(output) length (정확히는 encoder lstm의 유닛개수) : 64, 128 이 추천되며, p와 같을수록 좋다.
        p : decoder lstm feature(output) length (정확히는 decoder lstm의 유닛개수) : 64, 128 이 추천되며, p와 같을수록 좋다.
        target_len : 예측할 Y의 개수
        """
        self.encoder = Encoder(T=T, n=n, m=m)
        self.decoder = Decoder(p=p, m=n, n_dec=n)
        self.tail = DecoderTail(n+p, target_len)

    def forward(self, inp_data, return_inspection=False):
        """
        inp_data : batch, T, n
        """
        inp_norm = norm_x(inp_data)

        mask = torch.sum(torch.abs(inp_data), dim=2)
        mask = torch.where(mask > 0, 1.0, 0)  # batch, time

        enc_output, alpha = self.encoder(inp_norm)  # output : (batch, T, n)
        dec_output, beta, score_b = self.decoder(inp_norm, enc_output, mask)  # output : (batch_size, 1, m+p)

        output = dec_output.squeeze(dim=1)
        output = self.tail(output)

        if return_inspection:
            return output, alpha, beta, score_b  # (batch, target_len), (batch, T)
        return output


def norm_x(data):
    # data : (Batch, Time, Features)

    # time 0 ~ 5572
```

```
    # fp 0 ~ 96
    # fn 0 ~ 99
    # fl 0 ~ 100
    # md -35.23 ~ 19.78
    # psd 0 ~ 23.79
    # vfi 0 ~ 100
    # psd -54 ~ 37
    # tdv -38 ~ 25

    factors = [1/1000]  # time
    factors += [1/100, 1/100, 1/100]  # FP, FN, FL
    factors += [1/50, 1/50, 1/100]  # MD, PSD, VFI
    factors += [1/50 for i in range(54)]  # PDVs
    factors += [1/50 for i in range(54)]  # TDVs
    factors = torch.FloatTensor(factors).to(data.device)

    return torch.multiply(data, factors)


def norm_y(data):
    factors = [50., 50., 100.]  # MD, PSD, VFI
    factors += [50. for i in range(54)]  # TDVs
    factors = torch.FloatTensor(factors).to(data.device)
    return torch.multiply(data, factors)

```

```python
import openpyxl as xl
from datetime import datetime
import pickle
import csv
import torch
from torch.utils.data import Dataset

# ======================================================================
col_dict = {
        "EyeID": "A",
        "ExamDate": "F",
        "DOB": "D",
        "FP": "Q",
        "FN": "R",
        "FixLoss": "S",
        "FixTry": "T",
        "MD": "W",
        "PSD": "Y",
        "VFI": "AA",
        "PDV start": "CD",
        "THV start": "EF",
        "TDV start": "IJ"
    }

min_x_count = 3  # minimum input VF count
timestep_size = 70  # should be larger than min_x_count
mask_value = 0.

max_y_FP = 33  # max false positive (0~100)
max_y_FN = 33  # max false negative (0~100)
max_y_FL = 33  # max fix. loss (%) (0~100)
# ======================================================================

demo_header = ["EyeID", "Age", "X_cnt", "X_interval", "Y_interval", "Y_start_idx", "Y_MD", "X_Delta_MD"]


def str_to_number(col_str):
    """
    Excel의 컬럼 문자를 숫자 인덱스로 변환
    :param col_str: 엑셀의 컬럼 문자
    :return: 숫자 인덱스
    """
    col_str_upper = col_str.upper()
    ret = 0
    j = 1
    for i in range(len(col_str_upper)):
        ret += (ord(col_str_upper[-1 - i]) - 64) * j   # A = 65
        j *= 26
    return ret - 1


def make_xy(row_list, check_reliability=False, relative_exam_interval=True, date_ascending=True, empty_row_last=True):
    """
    row_list 에서 x,y 를 추출하여 리턴.
    :param row_list: 원본 VF 자료 리스트
    :param relative_exam_interval: True시 두 검사 날짜 간의 상대적 interval, False시 Y날짜로부터 절대적 interval
    :param check_reliability: 리턴되는 y 의 최소 신뢰도를 검사한다.
    :param date_ascending: True시 검사를 날짜 오름차순으로 정렬
    :param empty_row_last: True시 공백행을 맨 나중에 추가
```

```python
    :return:
    success, x, y, start_idx 순으로 리턴됨.
    """
    x = []
    y = None
    start_idx = None

    if row_list is None:
        return False, [], None, None

    # 음수 time interval 있는 경우가 있는 경우 제외
    for row in row_list:
        if row[0] < 0:
            return False, [], None, None

    # ==== start_idx 찾기 =====
    if check_reliability:
        for i in range(len(row_list)):
            if row_list[i][1] < max_y_FP and row_list[i][2] < max_y_FN and row_list[i][3] < max_y_FL:
                start_idx = i
                break
    else:
        start_idx = 0

    # start_idx 를 못찾았으면 실패
    if start_idx is None:
        return False, [], None, None

    # min_x_count 만큼도 없으면 실패
    if len(row_list)-start_idx-1 < min_x_count:
        return False, [], None, None

    y = row_list[start_idx][4:7] + row_list[start_idx][-54:]

    day_sum = 0
    for row in row_list[start_idx+1:]:
        if not relative_exam_interval:
            day_diff = row[0]
            row[0] += day_sum
            day_sum += day_diff
        if date_ascending:
            x.insert(0, row)  # 과거 --> 최근 순서 (ascending)
        else:
            x.append(row)  # 최근 --> 과거 순서 (descending)

    empty_row = list(mask_value for k in range(len(row_list[start_idx + 1])))
    for i in range(timestep_size-len(x)):
        if empty_row_last:
            x.append(empty_row)
        else:
            x.insert(0, empty_row)

    return True, x, y, start_idx


def open_excel_file(filepath, contains_header=True, check_reliability=False, max_row=0, relative_exam_interval=False, date_ascending=True, empty_row_last=True):
    # init output values
    x_return = []
    y_return = []
    demo_return = []

```

```python
    # read Excel file
    wb = xl.load_workbook(filepath, data_only=True, read_only=True)
    sheet = wb.worksheets[0]

    # init variables
    current_eye = ''
    prev_exam_date = None
    data_row_collection = []
    discard_count = 0
    age = 0
    for idx, excel_row in enumerate(sheet):
        # skip header row
        if idx == 0 and contains_header:
            continue
        print('reading row {}, current set count: {}, discarded: {}'.format(idx+1, len(y_return), discard_count))

        eye_id = excel_row[str_to_number(col_dict["EyeID"])].value.upper()

        # convert to sequential data, separate x, y, and save to return list
        if current_eye != eye_id:  # completed to read all VFs of one patient
            success, x, y, start_idx = make_xy(data_row_collection,
                                               check_reliability=check_reliability,
                                               relative_exam_interval=relative_exam_interval,
                                               date_ascending=date_ascending,
                                               empty_row_last=empty_row_last)
            if success:
                x_vf_cnt = len(data_row_collection) - 1 - start_idx  # 입력 시야 검사의 개수
                y_interval = data_row_collection[start_idx+1][0]  # 맨 마지막 시야 검사와 예측일 사이의 시간 간격 (day)
                x_interval = 0  # x 처음과 끝의 날짜 간격 차이
                for x_vf in data_row_collection[start_idx+2:]:
                    x_interval += x_vf[0]
                y_md = y[0]  # y 의 MD
                delta_x_md = data_row_collection[start_idx+1][4] - data_row_collection[-1][4]  # X 시작과 끝의 MD 차이
                demo_return.append([current_eye, age, x_vf_cnt, x_interval, y_interval, start_idx, y_md, delta_x_md])
                x_return.append(x)
                y_return.append(y)
                print('>>> set {} added. input VF count: {}'.format(len(x_return), x_vf_cnt))
            else:
                discard_count += 1
                print('>>> eye id {} discarded. input VF count: {}'.format(current_eye, len(data_row_collection)))
            if eye_id == 'LAST ROW' or eye_id == 'LASTROW' or eye_id == 'LAST_ROW':
                break
            current_eye = eye_id
            age = 0
            prev_exam_date = None
            data_row_collection = []

        # ==================== row parsing ===========================
        exam_date_cell = excel_row[str_to_number(col_dict["ExamDate"])]
        exam_date = exam_date_cell.value
        if not exam_date_cell.is_date:
            exam_date = datetime.strptime(exam_date_cell.value[0:10], '%Y-%m-%d')

        DOB_cell = excel_row[str_to_number(col_dict["DOB"])]
```

```python
        DOB = DOB_cell.value
        if not DOB_cell.is_date:
            try:
                DOB = datetime.strptime("{0:4d}-{1:02d}-{2:02d}".format(int(DOB.split('.')[0]), int(DOB.split('.')[1]), int(DOB.split('.')[2])), "%Y-%m-%d")
            except:
                DOB = None
        if age == 0 and DOB is not None:
            age = int((exam_date - DOB).days / 365)

        fix_loss_cnt = excel_row[str_to_number(col_dict["FixLoss"])].value
        fix_try_cnt = excel_row[str_to_number(col_dict["FixTry"])].value
        fix_loss_ratio = 0
        if fix_try_cnt > 0:
            fix_loss_ratio = fix_loss_cnt / fix_try_cnt
        reliability = [excel_row[str_to_number(col_dict["FP"])].value,  # FP %
                       excel_row[str_to_number(col_dict["FN"])].value,  # FN %
                       fix_loss_ratio * 100]  # Fix loss %
        if reliability[0] < 0:   # FP 가 음수인 경우가 있더라..
            reliability[0] = 0
        if reliability[1] < 0:   # FN 이 음수인 경우가 있더라..
            reliability[1] = 0

        global_idx = [excel_row[str_to_number(col_dict["MD"])].value,    # MD
                      excel_row[str_to_number(col_dict["PSD"])].value,   # PSD
                      excel_row[str_to_number(col_dict["VFI"])].value]   # VFI

        pdv_start = str_to_number(col_dict["PDV start"])
        pdv = [excel_row[i].value for i in range(pdv_start, pdv_start + 54)]
        pdv[26-1] = 0  # physiologic scotoma
        pdv[35-1] = 0  # physiologic scotoma

        thv_start = str_to_number(col_dict["THV start"])
        thv = [excel_row[i].value for i in range(thv_start, thv_start + 54)]

        tdv_start = str_to_number(col_dict["TDV start"])
        tdv = [excel_row[i].value for i in range(tdv_start, tdv_start + 54)]
        tdv[26-1] = 0  # physiologic scotoma
        tdv[35-1] = 0  # physiologic scotoma

        # ==================== x_row assignment ============================
        data_row = [0] if prev_exam_date is None else [(prev_exam_date - exam_date).days]
        prev_exam_date = exam_date
        data_row += reliability   # FP, FN, FL
        data_row += global_idx    # MD, PSD, VFI
        # data_row += thv
        data_row += pdv
        data_row += tdv
        data_row_collection.append(data_row)

        if idx > max_row > 0:
            print('max row count reached. reading terminated.')
            break

    return x_return, y_return, demo_return


def open_pickle(folder, name_tag="data"):
    print("Loading pickle files...")
    with open("{}₩₩pk_{}.pkl".format(folder, name_tag), "rb") as f:
        pkl = pickle.load(f)
    return pkl[0], pkl[1], pkl[2]  # x, y, demo
```

```


def save_pickle(x, y, demo, folder, name_tag="data"):
    with open("{}₩₩pk_{}.pkl".format(folder, name_tag), "wb") as f:
        pickle.dump([x, y, demo], f)


def save_as_csv(data, filepath):
    with open(filepath, 'w') as f:
        writer = csv.writer(f, lineterminator='₩n')  # 줄 바꿈 코드(₩n)을 지정
        writer.writerows(data)


class VFDataset(Dataset):
    def __init__(self, x_encoder, y, phase='train'):
        self.x_encoder = x_encoder
        self.y = y
        self.phase = phase

    def __len__(self):
        return len(self.y)

    def __getitem__(self, idx):
        return torch.FloatTensor(self.x_encoder[idx]), torch.FloatTensor(self.y[idx])

```
